# High-Frequency rTMS Improves Cognitive Deficits in APP/PS1 Mice with Attenuation of Ferroptosis-Related Oxidative Injury

**DOI:** 10.3390/brainsci16080868

**Published:** 2026-08-16

**Authors:** Boya Lu, Meng Zhang, Zihao Ren, Tianjiu Wang, Zixuan Wang, Chong Ding

**Affiliations:** 1 College of Health Sciences & Biomedical Engineering, Hebei University of Technology, Tianjin 300130, China; 202322902012@stu.hebut.edu.cn (B.L.); embed.zhang@djics.cn (M.Z.); 202322902007@stu.hebut.edu.cn (Z.R.); 202432903028@stu.hebut.edu.cn (T.W.); 202422902007@stu.hebut.edu.cn (Z.W.); 2 State Key Laboratory of Intelligent Power Distribution Equipment and System, Tianjin 300130, China; 3 Hebei Key Laboratory of Bioelectromagnetics and Neural Engineering, Tianjin 300130, China

**Keywords:** Alzheimer’s disease, APP/PS1 mice, rTMS, ferroptosis, oxidative stress, neuronal excitability, ion channels

## Abstract

**Highlights:**

**What are the main findings?**

- High-frequency rTMS improved cognitive performance and partially restored hippocampal DG granule cell excitability in APP/PS1 mice.
- rTMS was accompanied by partial normalization of voltage-gated Na^+^ and K^+^ current abnormalities and attenuation of ferroptosis-related oxidative and mitochondrial injury.

**What are the implications of the main findings?**

- The findings suggest that ferroptosis-related oxidative injury is associated with hippocampal neuronal dysfunction in APP/PS1 mice.
- rTMS may provide a multi-level neuroprotective profile involving behavioral, electrophysiological, biochemical, and mitochondrial improvements, consistent with an association between neurofunctional recovery and attenuation of ferroptosis-related oxidative injury.

**Abstract:**

**Background/Objectives**: Repetitive transcranial magnetic stimulation (rTMS) is a non-invasive neuromodulatory approach with potential therapeutic value for cognitive impairment in Alzheimer’s disease (AD). Ferroptosis-related oxidative injury has been implicated in AD-associated neuronal dysfunction, but whether rTMS-induced functional improvement is accompanied by changes in ferroptosis-related oxidative injury remains unclear. This study evaluated the effects of high-frequency rTMS on cognitive function, hippocampal neuronal excitability, and ferroptosis-related oxidative injury in amyloid precursor protein/presenilin-1 (APP/PS1) mice, using Ferrostatin-1 (Fer-1) as a pharmacological comparator. **Methods**: Six-month-old female mice were used, including age-matched C57BL/6J controls and APP/PS1 mice assigned to the AD + Sham, AD + rTMS, and AD + Fer-1 groups (*n* = 6 per group). After 14 days of intervention, cognitive performance was assessed using behavioral tests. Whole-cell patch-clamp recordings were performed in hippocampal dentate gyrus granule neurons to evaluate neuronal excitability and voltage-gated sodium (Na^+^) and potassium (K^+^) channel properties. Biochemical assays and transmission electron microscopy were used to assess oxidative, iron-related, and mitochondrial changes, and mitochondrial ultrastructure was examined in an independent cohort (*n* = 3 per group) using transmission electron microscopy. **Results**: Compared with AD + Sham mice, high-frequency rTMS improved cognitive performance, increased evoked action potential firing, lowered the elevated action potential threshold, partially restored voltage-gated Na^+^ and K^+^ current amplitudes, and accelerated recovery of Na^+^ currents from inactivation. Fer-1 produced partially overlapping, but not identical, effects across behavioral, electrophysiological, biochemical, and ultrastructural outcomes. Both interventions increased hippocampal glutathione (GSH) levels, reduced malondialdehyde (MDA) and total iron levels, partially restored superoxide dismutase (SOD) activity, and improved mitochondrial ultrastructure and reduced the prevalence of mitochondrial profiles with small cross-sectional areas. **Conclusions**: High-frequency rTMS improved cognitive and hippocampal neuronal outcomes in female APP/PS1 mice. These improvements were accompanied by biochemical and mitochondrial changes compatible with attenuation of ferroptosis-related injury. However, the findings do not establish ferroptosis inhibition as either necessary or sufficient for the effects of rTMS.

## 1. Introduction

Alzheimer’s disease (AD) is a progressive neurodegenerative disorder characterized by cognitive decline, memory impairment, and functional disability [1]. Despite extensive research, effective disease-modifying treatments remain limited, partly because AD involves multiple interacting pathological processes, including amyloid-β accumulation, tau pathology, oxidative stress, and neuroinflammation [2,3]. These pathological processes can converge on synaptic dysfunction, mitochondrial impairment, and neuronal network abnormalities, thereby contributing to neuronal failure and cognitive deterioration [4,5]. The hippocampus plays a critical role in learning and memory and is particularly vulnerable to AD-related pathological changes. Therefore, hippocampal dysfunction represents an important target for investigating AD-associated cognitive impairment and evaluating potential therapeutic interventions in AD models [6].

Ferroptosis is a regulated form of cell death characterized by iron-dependent lipid peroxidation and impaired antioxidant defense [7,8]. In neurons, iron dyshomeostasis can promote reactive oxygen species generation through Fenton chemistry, whereas disruption of glutathione metabolism and glutathione peroxidase 4 activity may lead to lipid peroxide accumulation, membrane damage, mitochondrial abnormalities, and neuronal dysfunction [9,10]. Ferroptosis-related alterations, including iron accumulation, lipid peroxidation, oxidative stress, and mitochondrial injury, have been associated with neurodegenerative diseases, including AD [11]. These findings suggest that ferroptosis-related oxidative injury may be associated with AD-related neuronal impairment and cognitive decline.

Repetitive transcranial magnetic stimulation (rTMS) is a non-invasive neuromodulatory technique that uses time-varying magnetic fields to induce electrical activity in neural tissue [12,13]. Previous studies have shown that rTMS can improve cognitive performance in patients with AD and animal models, potentially through multiple interacting mechanisms, including modulation of cortical and hippocampal excitability and plasticity, neurotrophic signaling, neurotransmitter regulation, glymphatic clearance, and broader neuronal network activity [14,15,16]. In an AD-related rat model, low-frequency rTMS increased hippocampal nerve growth factor, brain-derived neurotrophic factor (BDNF), and N-methyl-D-aspartate (NMDA) receptor levels and improved long-term potentiation and spatial memory [17]. Complementing these preclinical findings, Moscatelli et al. reported that high-frequency rTMS applied to the dorsolateral prefrontal cortex in healthy trained female participants was associated with improved attentional performance, shorter reaction time, and increased salivary Orexin-A levels [18]. Because that study involved healthy human participants and dorsolateral prefrontal cortex stimulation, whereas the present study involved female amyloid precursor protein/presenilin-1 (APP/PS1) mice and an animal stimulation paradigm, the comparison is conceptual rather than direct. These findings indicate that ferroptosis-related oxidative injury should be considered one potentially relevant component of the broader biological response to rTMS rather than the sole explanation for its cognitive effects. In APP/PS1 mice, rTMS has also been reported to improve cognitive deficits and alter hippocampal neuronal electrophysiological properties, including action potential firing and voltage-gated sodium (Na^+^) and potassium (K^+^) channel activity [19]. However, whether rTMS treatment is accompanied by changes in ferroptosis-related oxidative injury during the improvement of AD-associated cognitive and neuronal dysfunction remains unclear. Therefore, the present study examined whether high-frequency rTMS-induced cognitive and neuronal functional changes are accompanied by alterations in ferroptosis-related oxidative injury in APP/PS1 mice.

To address this question, Ferrostatin-1 (Fer-1), a commonly used pharmacological inhibitor targeting ferroptosis-related lipid peroxidation, was used as a comparator [20,21]. Fer-1 allowed comparison of rTMS with a pharmacological intervention targeting ferroptosis-related oxidative injury. However, because Fer-1 primarily limits lipid peroxidation and ferroptosis-related indicators are not fully specific to ferroptosis [22], this comparative design was intended to evaluate association and phenotypic similarity rather than to establish a definitive ferroptosis-specific causal pathway.

Accordingly, we applied a multi-level experimental design in APP/PS1 mice treated with high-frequency rTMS or Fer-1. At the behavioral level, cognitive performance was assessed to evaluate the effects of rTMS and Fer-1 on AD-like cognitive deficits. At the electrophysiological level, whole-cell patch-clamp recordings were performed in hippocampal dentate gyrus (DG) granule neurons to evaluate neuronal excitability and voltage-gated Na^+^ and K^+^ channel properties. Biochemical assays and transmission electron microscopy were used to assess ferroptosis-related oxidative injury and mitochondrial ultrastructure, respectively.

## 2. Materials and Methods

### 2.1. Experimental Animals and Design

Six-month-old female APP/PS1 double-transgenic mice and age-matched female C57BL/6J wild-type mice were obtained from Hengrong Biotechnology (Tianjin, China). APP/PS1 mice carry human APP and mutant PS1 transgenes on a C57BL/6 background and develop age-dependent AD-like pathological and cognitive phenotypes [23]. Only female mice were used in this study to reduce sex-related variability and because sex-dependent behavioral and amyloid-related pathological differences have been reported in APP/PS1 mice and other AD mouse models [24]. The estrous cycle was not monitored or experimentally controlled, and estrous stage was not used as an inclusion, exclusion, or stratification criterion. All experimental procedures were approved by the Institutional Animal Care and Use Committee of Hebei University of Technology (Protocol No. HEBUTACUC2024036). The reporting of this study followed the ARRIVE 2.0 guidelines.

After a 7-day acclimation period, mice were group-housed under controlled temperature conditions (24 ± 1 °C) on a 12 h light/12 h dark reverse cycle, with ad libitum access to standard chow and water. For the main experimental cohort, APP/PS1 mice were randomly assigned to three groups: sham-stimulated APP/PS1 mice (AD + Sham, *n* = 6), high-frequency rTMS-treated APP/PS1 mice (AD + rTMS, *n* = 6), and Fer-1-treated APP/PS1 mice (AD + Fer-1, *n* = 6). Age-matched C57BL/6J mice served as wild-type controls (Control, *n* = 6). These 24 mice constituted the main experimental cohort used for behavioral testing, electrophysiological recordings, and biochemical assays. An additional independent cohort of mice was used for transmission electron microscopy (TEM; *n* = 3 per group). No a priori sample-size calculation was performed. This study was designed as an exploratory preclinical investigation, with six animals per group included in the main experimental cohort.

APP/PS1 mice were allocated to the AD + Sham, AD + rTMS, and AD + Fer-1 groups using simple randomization. The allocation sequence was generated using the RAND function in Microsoft Excel by an investigator who was not involved in subsequent outcome assessment or statistical analysis. Each animal was assigned a unique identification code, and group information was concealed from the investigators responsible for outcome assessment. Investigators conducting behavioral testing, electrophysiological recordings and cell selection, biochemical assays, TEM image selection and mitochondrial delineation, and statistical analyses were blinded to group allocation. Group codes were revealed only after data collection and the planned analyses had been completed. Because of the nature of the interventions, the investigator administering rTMS, sham stimulation, or Fer-1 treatment could not be blinded to treatment allocation.

**Figure 1:**
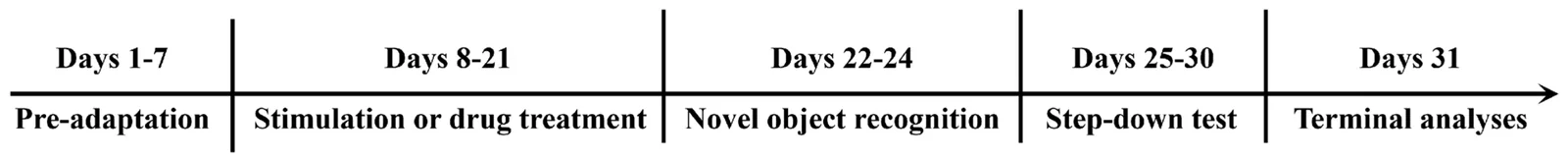
Overall experimental timeline.

Following the intervention period, all mice in the main experimental cohort underwent behavioral testing, including the novel object recognition (NOR) test and the step-down test (SDT). After completion of behavioral testing, the mice were euthanized for terminal tissue collection. Acute hippocampal slices were prepared for whole-cell patch-clamp recording, and the remaining hippocampal tissue that was not used for electrophysiological recording was immediately collected for biochemical assays of ferroptosis-related oxidative injury markers. Thus, electrophysiological and biochemical measurements were obtained from the same six mice per group using distinct tissue portions from each animal, and the mouse was treated as the biological experimental unit in both analyses. TEM was performed using a separate, independent cohort of mice that underwent the same intervention and behavioral-testing procedures as the main experimental cohort but did not contribute to the electrophysiological or biochemical analyses. Behavioral data from the TEM cohort were not included in the behavioral statistical analyses (Figure 1).

### 2.2. rTMS Treatment

A CCY-IA magnetic stimulation system with a Y064 circular animal coil was used for rTMS delivery (Yiruide Medical Equipment, Wuhan, China). The coil had an outer diameter of 56 mm and an inner diameter of 14 mm.

Before the formal intervention, the motor threshold (MT) was determined in 10 mice from the experimental cohort. These animals remained in the study after MT assessment and subsequently underwent their assigned interventions and outcome assessments. Briefly, mice were induced with 2% isoflurane (Beiyining; Qingdao Orbiepharm Co., Ltd., Qingdao, China) using a ZR-02 anesthesia machine (Shanghai Puxin Instrument Technology Co., Ltd., Shanghai, China) with 100% O_2_ as the carrier gas and maintained at 1% isoflurane. Light anesthesia was defined by absent spontaneous limb movement with preserved withdrawal to gentle paw pinching; minimum alveolar concentration (MAC) was not directly determined. The stimulation coil was positioned approximately 0.5 cm above the sagittal suture of the skull. Single-pulse stimulation was initiated at 1% stimulator output and increased in 1% increments while limb responses were monitored. The motor threshold (MT) was defined as the lowest intensity that evoked visible limb twitching in at least 5 of 10 consecutive pulses. Across 10 mice, the mean MT was 11.0 ± 0.7% of maximum stimulator output (range, 10–12%), and the intervention intensity was set at 80% MT [19,25].

**Figure 2:**
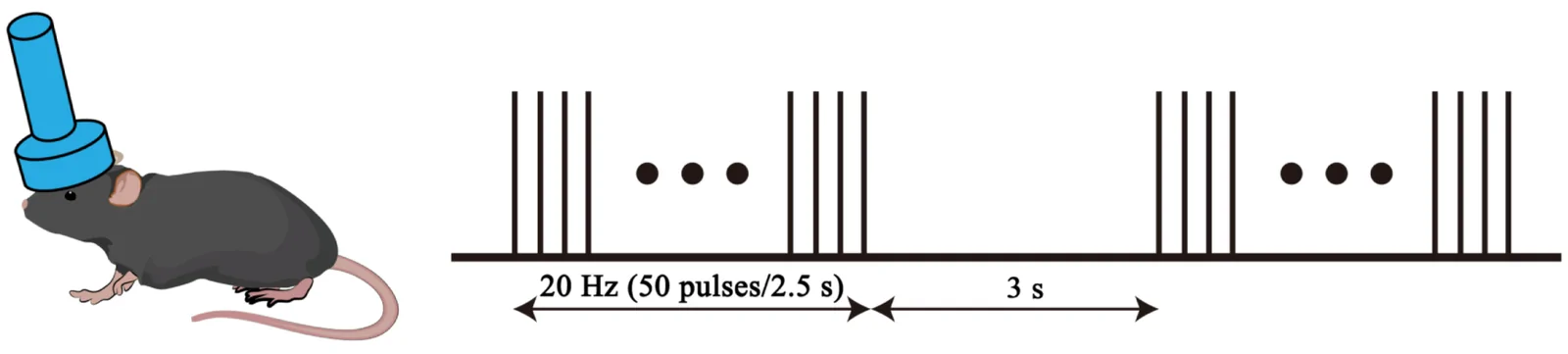
Schematic representation of the high-frequency rTMS protocol. Each train consisted of 50 pulses delivered at 20 Hz over 2.5 s, with a 3-s inter-train interval.

High-frequency rTMS was delivered at 20 Hz. Each daily stimulation session consisted of 10 trains, with each train containing 50 pulses over 2.5 s and an inter-train interval of 3 s. Thus, each mouse received 500 pulses per day, for a total of 7000 pulses over 14 consecutive days (Figure 2). During active stimulation, mice were manually restrained, and the coil was positioned parallel to and in close contact with the top of the head. Coil position was kept as consistent as possible across sessions. Because the 56-mm circular coil was large relative to the mouse head, stimulation was considered non-focal and broadly distributed rather than selectively targeted to a specific brain region. Accordingly, the hippocampus was treated as an outcome-assessment region rather than a selectively stimulated anatomical target.

Mice in the Control and AD + Sham groups underwent the same handling and restraint procedures on the same schedule. For sham stimulation, the coil was inverted vertically and positioned away from the head, allowing the animals to hear the stimulation sound while reducing effective magnetic-field exposure. Magnetic-field exposure at the position of the mouse head was not directly measured under the sham condition. Therefore, although the sham procedure reproduced the auditory component of stimulation, it may not have fully reproduced the cutaneous sensation, mechanical vibration, or residual magnetic exposure associated with active rTMS.

### 2.3. Administration of Fer-1

Ferrostatin-1 (Fer-1; MedChemExpress, Monmouth Junction, NJ, USA) was used as a pharmacological comparator targeting lipid peroxidation associated with ferroptosis-related injury [20,21]. Fer-1 was dissolved in a vehicle containing 2% dimethyl sulfoxide (DMSO), 50% polyethylene glycol 400 (PEG 400), 5% Tween-80, and sterile double-distilled water to a final concentration of 5 mg/mL.

Mice in the AD + Fer-1 group received Fer-1 by intraperitoneal injection at 2 mg/kg/day for 14 consecutive days. The injection volume was calculated individually according to body weight and Fer-1 concentration: injection volume (mL) = body weight (kg) × 2 mg/kg ÷ 5 mg/mL. Mice in the Control, AD + Sham, and AD + rTMS groups received an equivalent volume of the same vehicle by intraperitoneal injection once daily for 14 consecutive days. Fer-1 and vehicle administration were synchronized with the 14-day rTMS intervention schedule.

### 2.4. NOR

Object recognition memory was evaluated using the novel object recognition (NOR) paradigm in a 40 cm × 40 cm × 40 cm open-field arena with video recording. Mouse movements were tracked using the Any-maze system, with the head position used as the tracking target. Object exploration was defined as entry of the mouse’s head into the preset exploration zone. Climbing onto an object or upward exploration was not counted.

**Figure 3:**
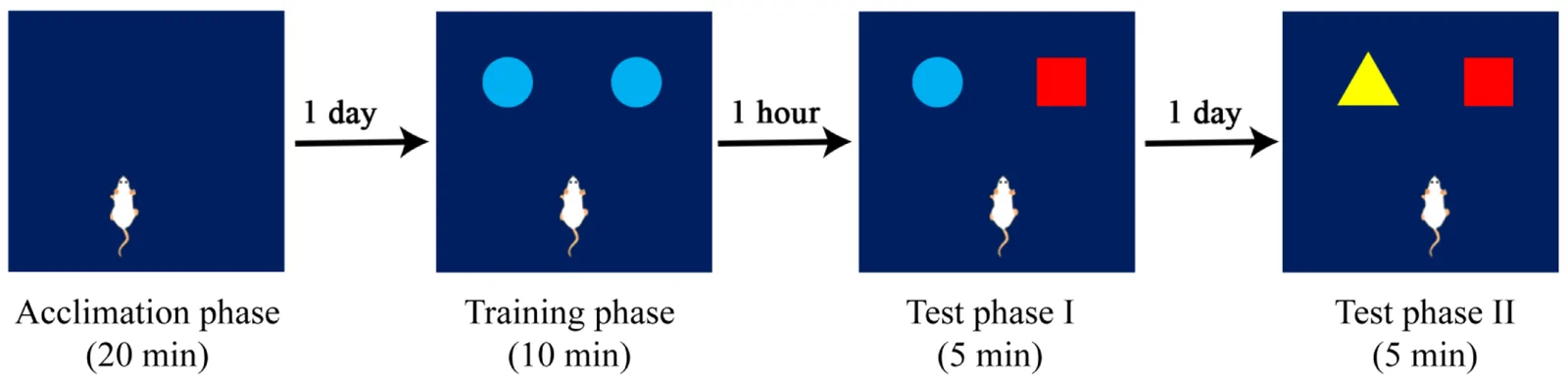
Schematic representation of the NOR test procedure.

The NOR test consisted of four phases (Figure 3). During adaptation, each mouse explored the empty arena for 20 min. Twenty-four hours later, two identical blue cylinders were placed in adjacent positions during the training phase, and each mouse was allowed to explore for 10 min.

Test Phase I was conducted 1 h after training to assess short-term recognition memory. The blue cylinder on the right side was replaced by a red cube, whereas the remaining blue cylinder served as the familiar object. Mice were allowed to explore for 5 min, and object exploration time was recorded.

Test Phase II was conducted 24 h after Test Phase I as part of a sequential object-replacement paradigm. The remaining original blue cylinder was replaced by a yellow triangular object, while the red cube introduced during Test Phase I remained in the arena. Because the mice had previously encountered the red cube, it was treated as the familiar object, whereas the yellow triangular object was treated as novel. Thus, Test Phase II assessed recognition following sequential object exposure rather than independent 24-h retention of the original training objects. Mice were again allowed to explore for 5 min.

During both test phases, time spent exploring the novel and familiar objects was designated as T1 and T2, respectively. In Test Phase I, T1 represented exploration of the red cube and T2 the remaining blue cylinder; in Test Phase II, T1 represented the yellow triangular object and T2 the previously presented red cube. Recognition performance was expressed as the cognitive index (CI): CI = T1/(T1 + T2). The identities and positions of the novel objects were kept constant across animals and were not counterbalanced, and no preliminary object-preference test was performed. Total object exploration time was calculated as T1 + T2. No predefined minimum-exploration exclusion criterion was applied, and all animals were included in the analysis.

### 2.5. SDT

Aversive learning and passive avoidance memory were assessed using an 8-channel automated SDT system (Chengdu Taimeng Technology Co., Ltd., Chengdu, China). The apparatus consisted of a controller, an electrical stimulator, and eight independent testing chambers. Each chamber measured 140 mm × 155 mm × 300 mm and contained a cylindrical insulated platform (45 mm in diameter and 45 mm in height) in the center. The floor consisted of a copper grid formed by 24 parallel copper rods.

**Figure 4:**
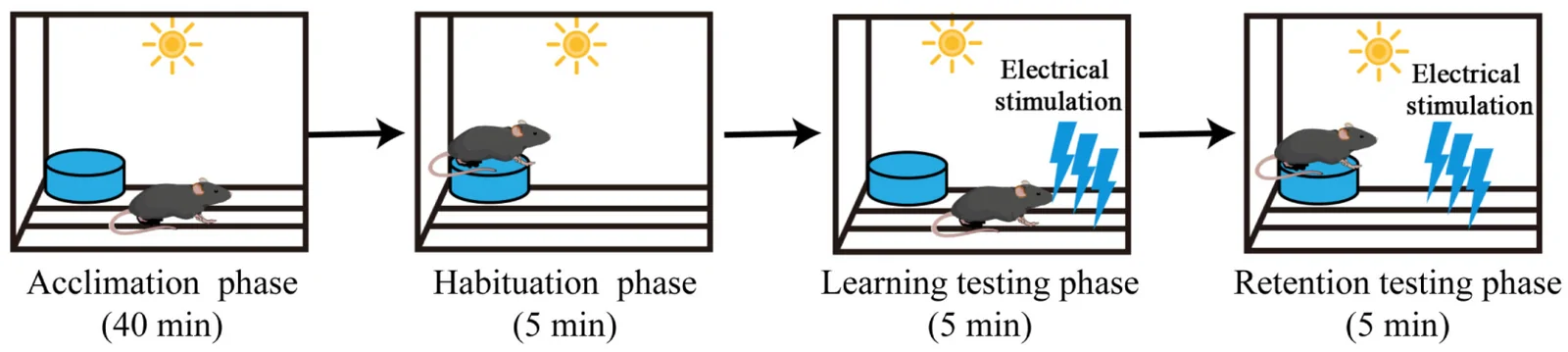
Step-down test procedure.

The SDT consisted of three phases: adaptation, electric-shock learning, and passive avoidance retention testing (Figure 4). During adaptation, each mouse was placed individually on the insulated platform facing the chamber wall and allowed to explore freely and move between the platform and grid floor for 5 min. No electrical stimulation was applied during this phase.

The electric-shock learning phase was conducted 24 h after adaptation. Each mouse was individually placed on the copper grid floor, which was energized at a nominal setting of 28 V. According to the equipment specifications, the electrical stimulus was delivered as an alternating current. The current at the animal–grid interface was not directly measured and may have varied with contact resistance. The grid remained energized throughout the 5-min learning period, and electrical stimulation occurred whenever the animal contacted the grid and ceased when the animal escaped onto the insulated platform. Timing began when the mouse first escaped to the platform. Each subsequent descent from the platform and contact with the grid was counted as a step-down error, and the percentage of time spent on the platform during the 5-min session was calculated.

The passive avoidance retention test was performed 24 h after the electric-shock learning phase. Each mouse was placed on the insulated platform facing the chamber wall, and the time to first step down from the platform was recorded as the passive avoidance latency. A maximum latency of 300 s was assigned if the mouse remained on the platform throughout the 5-min test period. The chamber and platform were cleaned with 70% ethanol between phases and animals to reduce potential olfactory interference.

### 2.6. Whole-Cell Patch-Clamp Recording

Whole-cell patch-clamp experiments were carried out on acute hippocampal slices prepared after the completion of behavioral tests. Mice were anesthetized with 4% chloral hydrate at 0.3 mL/100 g body weight. Adequate anesthesia was verified by the absence of the righting reflex and corneal reflex. Animals were then euthanized by cervical dislocation and rapidly decapitated. The brain was removed immediately and transferred into ice-cold oxygenated cutting solution. Brain isolation was completed within approximately 120 s to reduce ischemia- and hypoxia-related tissue damage.

The brain was mounted on the stage of a Leica VT1000S vibratome (Leica Biosystems, Deer Park, IL, USA), and 300-μm coronal sections containing the hippocampus were prepared. Slices with well-preserved hippocampal morphology were transferred to a holding chamber containing oxygenated artificial cerebrospinal fluid (ACSF). The ACSF was continuously saturated with 95% O_2_/5% CO_2_, and the chamber temperature was maintained at 30 °C. Slices were recovered for 60 min before electrophysiological recording.

Patch pipettes were fabricated from borosilicate glass capillaries using a Sutter micropipette puller. The capillaries had an outer diameter of 1.5 mm and an inner diameter of 0.86 mm. After filling with the corresponding intracellular solution, pipette resistance was 5–10 MΩ, with a tip diameter of approximately 1–3 μm. Pipettes were inspected to ensure that no air bubbles were present at the tip.

Slices were placed in a recording chamber on an upright microscope containing oxygenated extracellular recording solution. Whole-cell recordings were performed at 20 °C in a static bath without continuous superfusion. Electrophysiological signals were recorded using an EPC-10 dual-channel patch-clamp amplifier (HEKA Elektronik Dr. Schulze GmbH, Lambrecht, Germany), acquired using PatchMaster software (version v2x90.5, HEKA, Germany), and sampled at 20 kHz. The amplifier bandwidth parameter stored in the acquisition metadata was 100 kHz. DG granule cells were selected under infrared differential interference contrast optics based on their anatomical location and healthy appearance. Whole-cell recordings were accepted only from cells with stable electrophysiological properties, including a membrane capacitance of 6–10 pF and an input resistance of 150–300 MΩ. After membrane rupture, establishment of the whole-cell recording configuration was confirmed by a marked decrease in membrane resistance and stabilization of the electrophysiological signals. Slow-capacitance and series-resistance compensation were subsequently applied, with the compensation level set to at least 80%. Recordings showing an unstable baseline, excessive noise, evident resistance instability, or loss of the whole-cell configuration were excluded. A predefined numerical cutoff for the maximum allowable change in series resistance was not documented in the original recording protocol [19]. Brain slices and recording files were identified using coded labels. Cell selection, electrophysiological recording, and offline data analysis were performed by investigators blinded to treatment allocation. Group codes were revealed only after the electrophysiological data analysis had been completed.

In current-clamp mode, resting membrane potential (RMP) was measured over a 1000 ms recording period. Action potential firing was evoked by injecting a +100 pA depolarizing current pulse for 500 ms, and the number of elicited action potentials was quantified. For single action potential analysis, waveform parameters were measured within a 25 ms time window, including threshold potential, peak potential, half-width, maximum rising slope, and maximum falling slope [25].

In voltage-clamp mode, voltage-gated Na^+^ and K^+^ currents were recorded from DG granule neurons. For Na^+^ current measurements, cells were maintained at a holding potential of −70 mV, followed by 200-ms depolarizing voltage commands ranging from −70 mV to +40 mV in 10-mV increments. The Na^+^ current was pharmacologically isolated using an extracellular solution containing blockers of K^+^ and calcium (Ca^2+^) channels and was characterized as a tetrodotoxin-sensitive inward current.

For K^+^ current analysis, transient outward K^+^ current (*I*_A_) and delayed rectifier K^+^ current (*I*_K_) were measured using separate recording protocols. *I*_A_ was obtained in the presence of tetrodotoxin, cadmium chloride (CdCl_2_), and tetraethylammonium chloride (TEA-Cl), which were used to suppress Na^+^ currents, Ca^2+^ currents, and delayed rectifier K^+^ currents, respectively. *I*_K_ was recorded with tetrodotoxin, CdCl_2_, and 4-aminopyridine (4-AP) in the extracellular solution to inhibit Na^+^ currents, Ca^2+^ currents, and transient outward K^+^ currents, respectively [19].

Current–voltage curves were constructed using membrane potential as the x-axis and peak current amplitude as the y-axis. For activation curves, peak currents were converted to conductance using the following equation:

*G* = *I*/(*V_m_* − *V*_rev_), (1)

where *I* is the peak current, *V_m_* is the test membrane potential, *V*_rev_ is the reversal potential, and *G* is the conductance. Normalized conductance was fitted with the Boltzmann equation:

*G*/*G*_max_ = 1/{1 + exp[(*V_m_* − _1/2_)/*k*]}, (2)

where _1/2_ is the half-activation voltage and *k* is the slope factor. For inactivation curves, *I*/*I*_max_ was plotted against the pre-pulse voltage and fitted with the Boltzmann equation:

*I*/*I*_max_ = 1/{1 + exp[(*V_m_* − _1/2_′)/*k*_1_]}, (3)

where _1/2_′ is the half-inactivation voltage and *k*_1_ is the slope factor. Recovery from inactivation was analyzed using paired-pulse protocols, and the recovery curve was fitted with a single-exponential equation:

*I*_2_/*I*_1_ = *A* + *B* exp(−*t*/*τ*), (4)

where *I*_1_ and *I*_2_ represent the peak currents evoked by the conditioning and test pulses, respectively; *t* is the inter-pulse interval, *τ* is the recovery time constant, and A and B are constants.

The compositions of the cutting solution, ACSF, extracellular solutions, and intracellular solutions are listed in Table 1.

### 2.7. Biochemical Assays of Ferroptosis-Related Oxidative Injury Markers

Following behavioral testing, the hippocampal tissue remaining after preparation of acute slices for whole-cell patch-clamp recording was collected for biochemical analysis. Biochemical and electrophysiological measurements therefore came from the same six mice per group using distinct tissue portions, with the mouse treated as the biological experimental unit. The collected tissue was processed on ice, snap-frozen in liquid nitrogen, and stored at −80 °C until analysis.

Approximately 0.1 g of wet hippocampal tissue from each mouse was homogenized on ice in 1 mL of the extraction buffer supplied with the corresponding assay kit. The homogenates were centrifuged according to the manufacturers’ protocols, and the resulting supernatants were used to measure reduced glutathione (GSH), malondialdehyde (MDA), superoxide dismutase (SOD), total iron, ferrous iron (Fe^2+^), and ferric iron (Fe^3+^). Commercial assay kits were purchased from Beijing Solarbio Science & Technology Co., Ltd. (Beijing, China; BC1170, BC0025, BC4355, BC5415, and BC0175). Results were normalized to wet tissue mass rather than total protein concentration.

Each mouse contributed one biological sample, which was assayed once for each biochemical outcome and used as the animal-level value. No technical replicate measurements were performed; therefore, intra-assay coefficients of variation could not be estimated from the study samples. No measurements were identified as being below the assay detection limits in the available experimental records. These biochemical indices were interpreted as markers of ferroptosis-related oxidative injury rather than ferroptosis-specific markers [22].

### 2.8. Transmission Electron Microscopy

Transmission electron microscopy (TEM) was performed using an independent cohort of three mice per group that underwent the same intervention and behavioral-testing procedures as the main experimental cohort but did not contribute to the behavioral statistical analyses, electrophysiological recordings, or biochemical assays. TEM was used to examine mitochondrial ultrastructure in hippocampal dentate gyrus (DG) neurons. After completion of the intervention and behavioral testing, mice assigned to TEM analysis were anesthetized and rapidly decapitated. The brain was quickly removed, and hippocampal tissue samples containing the DG region were dissected, immediately immersed in 2.5% glutaraldehyde, and fixed for 2–4 h.

After primary fixation, samples were washed with phosphate-buffered saline (PBS) and dehydrated through a graded ethanol series from 70% to 100%, with each dehydration step lasting 20 min. The dehydrated samples were infiltrated with resin and then embedded and polymerized. Ultrathin sections were cut using an ultramicrotome at a thickness of 50–100 nm. Sections were stained with heavy metal salts to enhance mitochondrial contrast.

The stained ultrathin sections were placed on copper grids and examined using a transmission electron microscope (JEM-1200X, Shimadzu, Kyoto, Japan). Images were obtained from comparable anatomical locations within the hippocampal DG region. For quantitative morphometric analysis, non-overlapping fields containing clearly identifiable neuronal somata and perinuclear cytoplasm with adequately preserved ultrastructure were selected. Fields containing obvious sectioning artifacts, tissue disruption, or insufficient image contrast were excluded. All images included in the quantitative analysis were acquired at the same magnification and at the 2 μm imaging scale. Images acquired at the 200 nm scale were used only for qualitative visualization of mitochondrial membranes and cristae and were not included in the morphometric analysis.

TEM grids and images were identified using coded labels. Image acquisition, field selection, and manual delineation of mitochondrial profiles were performed by an investigator blinded to group allocation. Group codes were revealed only after morphometric measurements were completed. Three mice per group were analyzed, with ten non-overlapping fields examined per mouse at the 2 μm imaging scale. Mitochondrial profiles were manually outlined using the Freehand Selection tool in ImageJ (version 1.54u) after scale calibration, and the mitochondrial cross-sectional area was recorded using the Area parameter. At least four mitochondrial profiles were measured per image.

For each mouse, measurements from the ten images were combined to calculate the animal-level percentages of mitochondrial profiles with cross-sectional areas < 5 μm^2^ and <10 μm^2^. These animal-level percentages were used for inferential statistical analysis, with the mouse treated as the experimental unit (*n* = 3 mice per group). Group-level frequency distributions of individual mitochondrial cross-sectional areas were presented for descriptive purposes only. Individual mitochondrial profiles, images, and frequency-distribution bins were not treated as independent experimental units.

### 2.9. Data Analysis

Statistical analysis was conducted using Origin 2018 and GraphPad Prism 8.1. All analyses were conducted using coded group identifiers, and group allocation was revealed only after completion of the planned analyses. Unless otherwise stated, data are presented as mean ± standard error of the mean (SEM), with statistical significance defined as *p* < 0.05. Prior to group comparisons, the Shapiro–Wilk test was used to examine data normality, and Levene’s test was applied to evaluate homogeneity of variance. Given the limited sensitivity of formal assumption tests with the small group sizes used in this study, these tests were supplemented by graphical inspection of model residuals, including quantile–quantile (Q-Q) plots and residual-versus-fitted plots, where applicable. Parametric statistical methods were used only when the distributional assumptions were considered reasonably satisfied on the basis of both formal tests and graphical inspection.

For passive avoidance latency, the number of observations reaching the 300-s cutoff was examined to assess potential ceiling effects. Normality was evaluated using the Shapiro–Wilk test together with inspection of the data distribution, and homogeneity of variance was assessed using Levene’s test. Because no substantial ceiling effect was observed and the assumptions of normality and homogeneity of variance were satisfied, passive avoidance latency was analyzed using one-way ANOVA followed by Tukey’s post-hoc test.

For single-endpoint outcomes, including behavioral measurements, electrophysiological variables, biochemical indices, fitted ion-channel kinetic parameters, and mitochondrial morphometric measurements, comparisons among groups were analyzed by one-way analysis of variance (ANOVA). When appropriate, Tukey’s post-hoc test was performed for multiple comparisons. Treatment group was set as the independent factor, with four levels: Control, AD + Sham, AD + rTMS, and AD + Fer-1.

For the action-potential analyses, the mouse was treated as the experimental unit. Two cells were recorded from each mouse, and the corresponding cell-level measurements were averaged within each animal before statistical analysis. Therefore, each mouse contributed one animal-level value for each action-potential endpoint, and group comparisons were performed using *n* = 6 animal-level observations per group.

For current–voltage (*I*-*V*) curve analyses of voltage-gated Na^+^ currents, transient outward K^+^ currents (*I*_A_), and delayed rectifier K^+^ currents (*I*_K_), two-way repeated-measures ANOVA was performed, with treatment group as the between-subject factor and test membrane potential as the within-cell factor. The Geisser–Greenhouse correction was applied where appropriate to account for potential violations of sphericity. If a significant main effect or interaction was identified, Tukey’s post-hoc test was applied for post-hoc multiple comparisons. For ion-current analyses, one cell from each of six mice per group was analyzed. No other parameters were analyzed by two-way ANOVA unless specifically stated. The number of animals, recorded cells, analyzed images, or biological replicates for each experiment is provided in the corresponding figure legends. To complement *p* values, effect sizes and confidence intervals were also reported.

For TEM morphometric analysis, the mouse was treated as the experimental unit. Mitochondrial profiles measured across ten images from the same mouse were combined to calculate one animal-level percentage for each predefined cross-sectional-area threshold. These animal-level values were analyzed by one-way ANOVA followed by Tukey’s post-hoc test. The group-level frequency distributions of individual mitochondrial cross-sectional areas were considered descriptive only, and no inferential statistical tests were performed using individual mitochondria, images, or frequency-distribution bins as independent observations.

To facilitate interpretation beyond statistical significance alone, exact *p* values were reported whenever possible, together with the corresponding effect sizes and confidence intervals. Partial eta squared (ηp^2^) was used as the effect-size measure for the main effects and interactions obtained from the one-way and two-way ANOVA models. For relevant post-hoc comparisons, Tukey-adjusted between-group mean differences and their two-sided 95% confidence intervals (CIs) were reported. Effect-size estimates, confidence intervals, and *p* values were considered collectively when interpreting the magnitude and precision of the observed differences.

No formal hierarchy of primary and secondary outcomes was prospectively prespecified because this study was designed as an exploratory preclinical investigation examining behavioral, electrophysiological, biochemical, and ultrastructural outcomes across multiple biological levels. Tukey’s post-hoc procedure was used to control multiplicity among pairwise group comparisons within each individual endpoint. No additional global multiplicity correction was applied across the heterogeneous family of endpoints. Accordingly, analyses across the multiple behavioral, electrophysiological, biochemical, kinetic, and morphometric outcomes were considered exploratory, and the findings were interpreted on the basis of the overall pattern of evidence together with exact *p* values, effect sizes, and 95% CIs rather than statistical significance alone.

## 3. Results

### 3.1. Effects of rTMS and Fer-1 on Cognitive Function

#### 3.1.1. Effects of rTMS and Fer-1 on NOR

**Figure 5:**
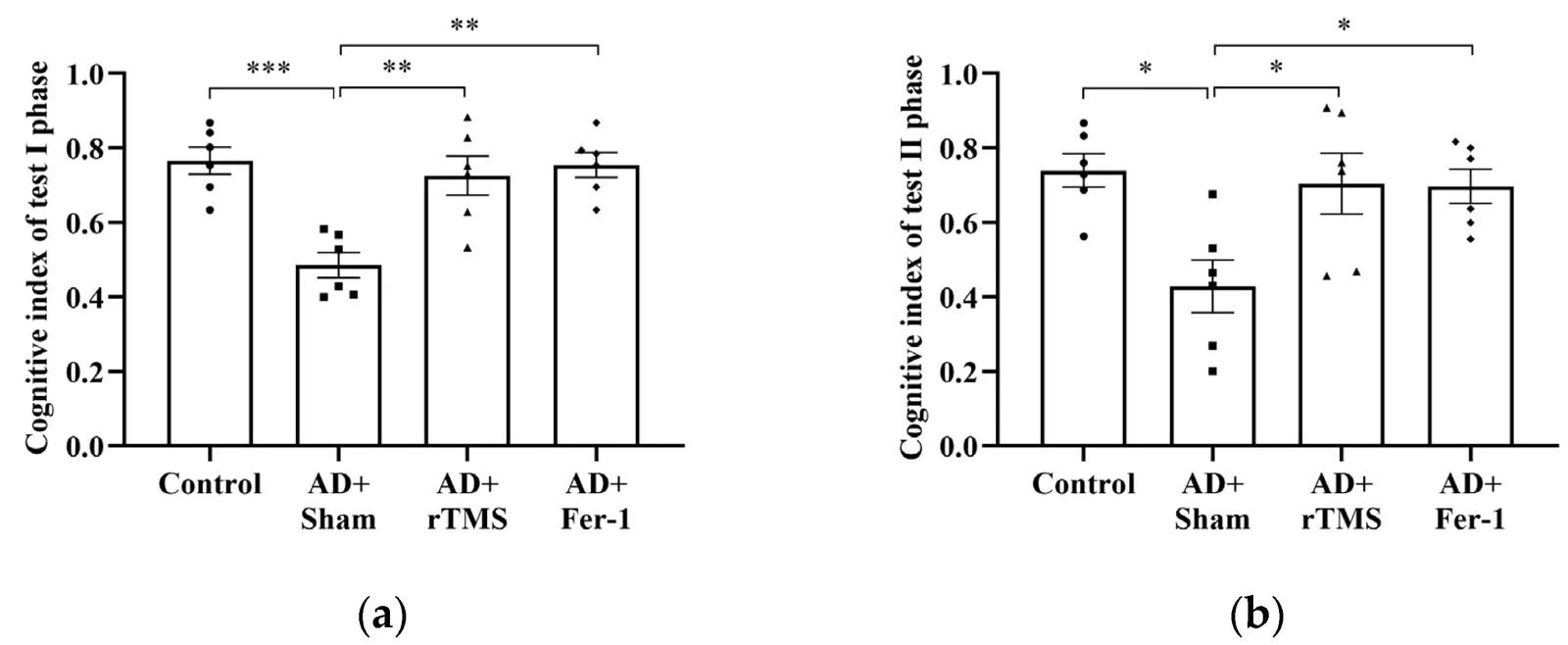
Cognitive index in the novel object recognition (NOR) test. (**a**) Test Phase I. (**b**) Test Phase II. Data are presented as mean ± SEM from six mice per group, with the mouse treated as the experimental unit. Statistical comparisons were performed using one-way ANOVA followed by Tukey’s post-hoc test. Significance symbols indicate the Tukey-adjusted between-group comparisons shown in the figure: * *p* < 0.05, ** *p* < 0.01, *** *p* < 0.001; *n* = 6.

Object recognition memory was assessed using the NOR test. In Test Phase I, the cognitive index differed significantly among groups (*F*(3, 20) = 11.11, *p* = 1.65 × 10^−4^, partial η^2^ = 0.625). Compared with the Control group, the AD + Sham group showed a 0.280 lower cognitive index (Tukey-adjusted 95% CI, 0.123 to 0.438; adjusted *p* = 3.92 × 10^−4^). Relative to AD + Sham, the cognitive index was increased by 0.240 in the AD + rTMS group (95% CI, 0.083 to 0.398; adjusted *p* = 0.603 × 10^−4^) and by 0.270 in the AD + Fer-1 group (95% CI, 0.112 to 0.427; adjusted *p* = 0.000603; Figure 5a). The AD + rTMS and AD + Fer-1 groups did not differ significantly (mean difference, −0.029; 95% CI, −0.187 to 0.128; adjusted *p* = 0.954).

In Test Phase II, the cognitive index also differed significantly among groups (*F*(3, 20) = 5.24, *p* = 0.00782, partial η^2^ = 0.440). Compared with the Control group, the AD + Sham group showed a 0.311 lower cognitive index (Tukey-adjusted 95% CI, 0.063 to 0.559; adjusted *p* = 0.011). Relative to AD + Sham, the cognitive index was increased by 0.276 in the AD + rTMS group (95% CI, 0.027 to 0.524; adjusted *p* = 0.0262) and by 0.268 in the AD + Fer-1 group (95% CI, 0.020 to 0.517; adjusted *p* = 0.031; Figure 5b). The AD + rTMS and AD + Fer-1 groups did not differ significantly (mean difference, 0.008; 95% CI, −0.241 to 0.256; adjusted *p* = 0.999).

Total object exploration time did not differ significantly among groups during Test Phase I or Test Phase II (both *p* > 0.05), suggesting that the differences in cognitive index were unlikely to be explained solely by differences in overall object exploration.

These results indicate impaired object recognition performance in APP/PS1 mice, whereas high-frequency rTMS and Fer-1 improved recognition performance across both test phases.

#### 3.1.2. Effects of rTMS and Fer-1 on SDT

**Figure 6:**
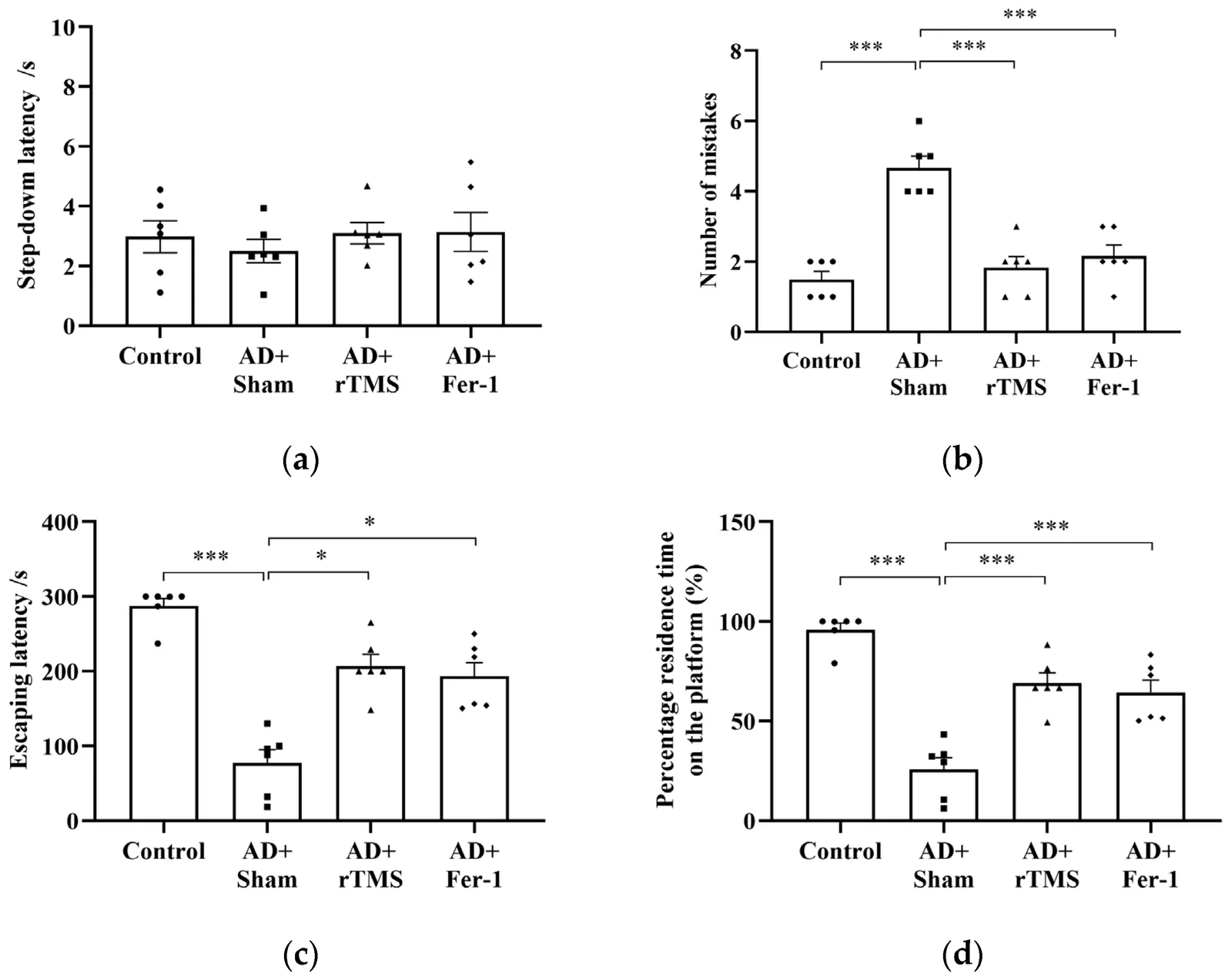
Step-down test results. (**a**) Step-down latency. (**b**) Number of mistakes. (**c**) Escape latency. (**d**) Percentage residence time on the platform. Data are presented as mean ± SEM from six mice per group, with the mouse treated as the experimental unit. Statistical comparisons were performed using one-way ANOVA followed by Tukey’s post-hoc test. Significance symbols indicate the Tukey-adjusted between-group comparisons shown in the figure: * *p* < 0.05, *** *p* < 0.001; *n* = 6.

The step-down test was used to evaluate aversive learning and passive avoidance memory. During the adaptation phase, step-down latency did not differ significantly among the Control, AD + Sham, AD + rTMS, and AD + Fer-1 groups (*F*(3, 20) = 0.30, *p* = 0.825), indicating comparable baseline exploratory and motor performance before conditioning (Figure 6a).

During the learning phase, the number of step-down errors differed significantly among groups (*F*(3, 20) = 23.78, *p* = 8.38 × 10^−7^, partial η^2^ = 0.781). Compared with the Control group, the AD + Sham group showed 3.17 more step-down errors (Tukey-adjusted 95% CI, 2.00 to 4.34; adjusted *p* = 1.53 × 10^−6^). Relative to AD + Sham, the number of errors was reduced by 2.83 in the AD + rTMS group (95% CI, −4.00 to −1.66; adjusted *p* = 7.72 × 10^−6^) and by 2.50 in the AD + Fer-1 group (95% CI, −3.67 to −1.33; adjusted *p* = 4.22 × 10^−5^; Figure 6b). The AD + rTMS and AD + Fer-1 groups did not differ significantly (mean difference, −0.33; 95% CI, −1.50 to 0.84; adjusted *p* = 0.855).

Only three animals reached the 300-s cutoff, and no substantial ceiling effect was observed. In the passive avoidance retention test, latency differed significantly among groups (*F*(3, 20) = 30.04, *p* = 1.32 × 10^−7^, partial η^2^ = 0.818). Compared with the AD + Sham group, latency was higher in the Control group (mean difference, 209.81 s; Tukey-adjusted 95% CI, 147.38 to 272.24; adjusted *p* = 5.01 × 10^−8^), AD + rTMS group (mean difference, 129.43 s; 95% CI, 66.99 to 191.86; adjusted *p* = 6.17 × 10^−5^), and AD + Fer-1 group (mean difference, 115.85 s; 95% CI, 53.42 to 178.29; adjusted *p* = 2.39 × 10^−4^; Figure 6c). The AD + rTMS and AD + Fer-1 groups did not differ significantly (mean difference, 13.57 s; 95% CI, −48.86 to 76.01; adjusted *p* = 0.928).

The percentage of time spent on the platform also differed significantly among groups (*F*(3, 20) = 30.04, *p* = 1.32 × 10^−7^, partial η^2^ = 0.818). Compared with the AD + Sham group, platform residence time was higher in the Control group (mean difference, 69.94 percentage points; Tukey-adjusted 95% CI, 49.13 to 90.75; adjusted *p* = 5.01 × 10^−8^), AD + rTMS group (mean difference, 43.14 percentage points; 95% CI, 22.33 to 63.95; adjusted *p* = 6.17 × 10^−5^), and AD + Fer-1 group (mean difference, 38.62 percentage points; 95% CI, 17.81 to 59.43; adjusted *p* = 2.39 × 10^−4^; Figure 6d). The AD + rTMS and AD + Fer-1 groups did not differ significantly (mean difference, 4.52 percentage points; 95% CI, −16.29 to 25.34; adjusted *p* = 0.928).

Together, these findings indicate impaired aversive learning and passive avoidance memory in APP/PS1 mice, both of which were improved by high-frequency rTMS and Fer-1.

### 3.2. Effects of rTMS and Fer-1 on Neuronal Function

#### 3.2.1. Effects of rTMS and Fer-1 on Action Potentials

**Figure 7:**
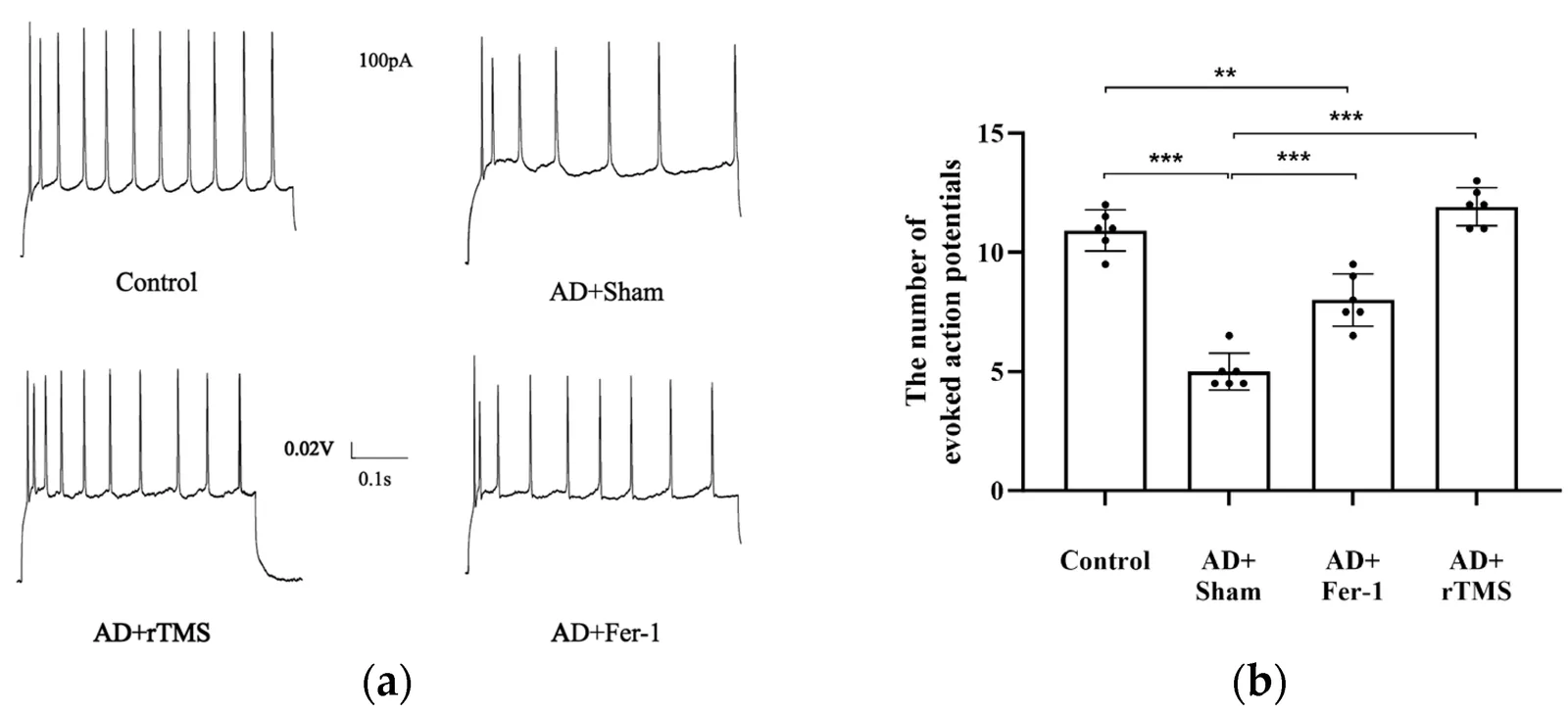
Action potential firing activity. (**a**) Representative AP traces evoked by a +100 pA depolarizing current injection for 500 ms. (**b**) Number of evoked APs. Data are presented as mean ± SEM from six mice per group. Two cells were recorded from each mouse, and the corresponding cell-level measurements were averaged to generate one animal-level value, with the mouse treated as the experimental unit. Statistical comparisons were performed using one-way ANOVA followed by Tukey’s post-hoc test. Significance symbols indicate the Tukey-adjusted between-group comparisons shown in the figure: ** *p* < 0.01, *** *p* < 0.001; *n* = 6.

Action potential firing evoked by a +100 pA depolarizing current injection was used to evaluate the excitability of hippocampal DG granule neurons. Animal-level analysis showed a large treatment effect on the number of evoked action potentials (*F*(3, 20) = 73.32, *p* = 5.76 × 10^−11^, partial η^2^ = 0.917). Compared with the Control group, the AD + Sham group generated 5.92 fewer APs (Tukey-adjusted 95% CI, −7.36 to −4.48; adjusted *p* = 1.68 × 10^−9^). Relative to AD + Sham, AP output was increased by 6.92 APs after rTMS (95% CI, 5.48 to 8.36; adjusted *p* = 1.05 × 10^−10^) and by 3.00 APs after Fer-1 treatment (95% CI, 1.56 to 4.44; adjusted *p* = 5.88 × 10^−5^; Figure 7). The Fer-1 group generated 3.92 fewer APs than the rTMS group (95% CI, −5.36 to −2.48; adjusted *p* = 1.43 × 10^−6^), consistent with an intermediate effect, whereas the rTMS and Control groups did not differ significantly (mean difference, 1.00 AP; 95% CI, −0.44 to 2.44; adjusted *p* = 0.243).

**Figure 8:**
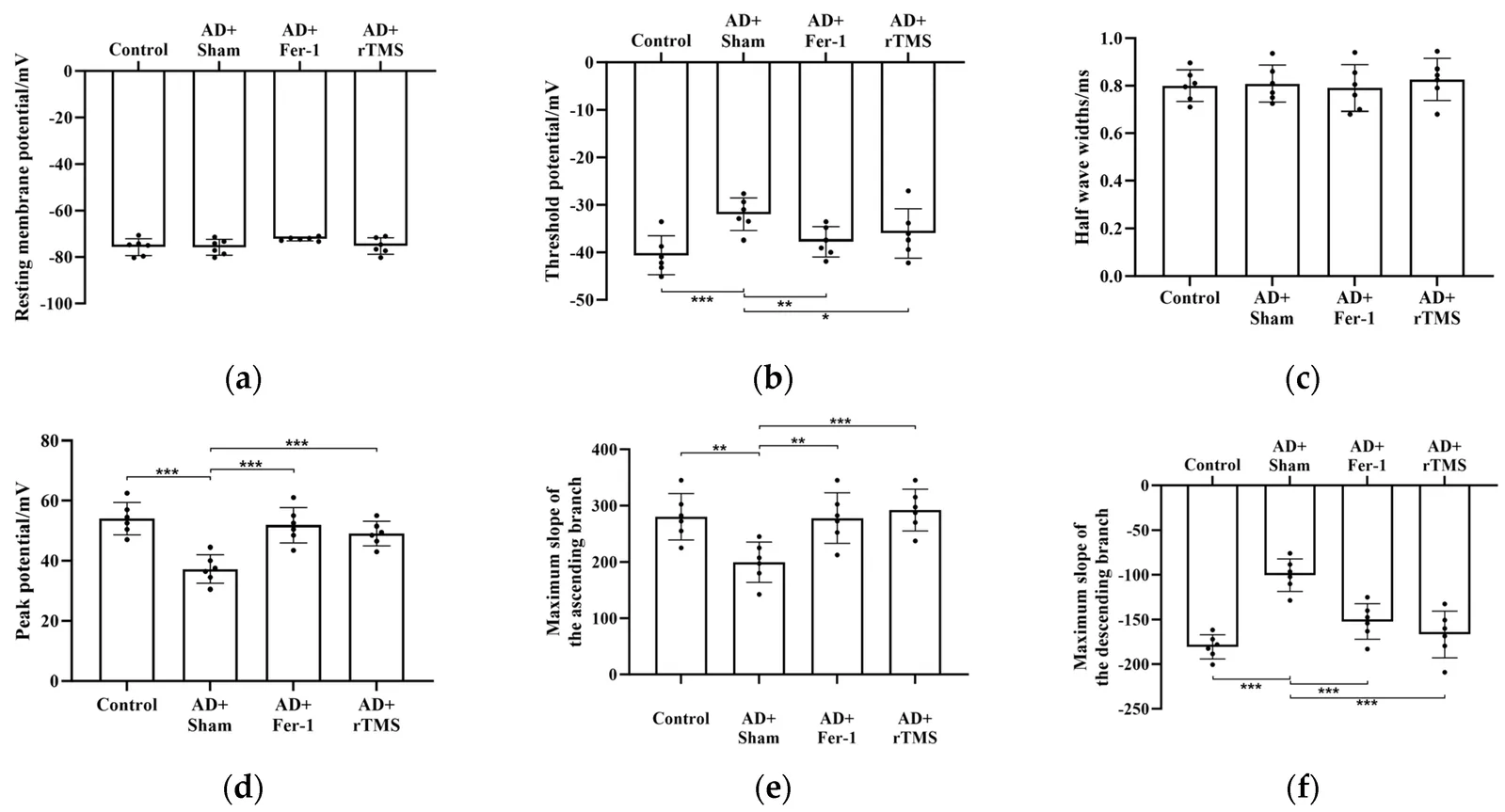
Neuronal excitability parameters. (**a**) RMP. (**b**) Threshold potential. (**c**) Half wave width. (**d**) AP peak potential. (**e**) Maximum slope of the ascending branch. (**f**) Maximum slope of the descending branch. Two cells were recorded from each mouse, and the corresponding cell-level measurements were averaged to generate one animal-level value, with the mouse treated as the experimental unit. Statistical comparisons were performed using one-way ANOVA followed by Tukey’s post-hoc test. Significance symbols indicate the Tukey-adjusted between-group comparisons shown in the figure: * *p* < 0.05, ** *p* < 0.01, *** *p* < 0.001; *n* = 6.

Resting membrane potential did not differ among groups at the animal level (*F*(3, 20) = 1.86, *p* = 0.168, partial η^2^ = 0.218; Figure 8a). The difference between the AD + Sham and Control groups was −0.05 mV (Tukey-adjusted 95% CI, −5.03 to 4.92; adjusted *p* = 0.99999), and none of the pairwise comparisons were significant. In contrast, AP threshold potential showed a large overall treatment effect (*F*(3, 20) = 12.57, *p* = 7.65 × 10^−5^, partial η^2^ = 0.653). Compared with the Control group, the AP threshold in the AD + Sham group was 9.50 mV more depolarized (Tukey-adjusted 95% CI, 4.87 to 14.13; adjusted *p* = 6.95 × 10^−5^), indicating that greater depolarization was required to initiate action potentials. Relative to AD + Sham, the threshold shifted in the hyperpolarizing direction by 7.48 mV after Fer-1 treatment (mean difference, −7.48 mV; 95% CI, −12.10 to −2.85; adjusted *p* = 0.00110) and by 7.03 mV after rTMS (mean difference, −7.03 mV; 95% CI, −11.66 to −2.41; adjusted *p* = 0.00202; Figure 8b).

AP half-width was similar across groups at the animal level (*F*(3, 20) = 0.20, *p* = 0.897, partial η^2^ = 0.029; Figure 8c). The mean difference between the AD + Sham and Control groups was 0.008 ms (Tukey-adjusted 95% CI, −0.127 to 0.144; adjusted *p* = 0.998), indicating little evidence of an effect on AP duration. In contrast, AP peak potential showed a large treatment effect (*F*(3, 20) = 12.77, *p* = 6.90 × 10^−5^, partial η^2^ = 0.657). Relative to the AD + Sham group, peak potential was higher by 16.75 mV in the Control group (95% CI, 8.48 to 25.02; adjusted *p* = 8.27 × 10^−5^), by 14.58 mV in the AD + Fer-1 group (95% CI, 6.31 to 22.85; adjusted *p* = 4.28 × 10^−4^), and by 11.75 mV in the AD + rTMS group (95% CI, 3.48 to 20.02; adjusted *p* = 0.00381; Figure 8d).

The maximum rising slope of APs also showed an overall treatment effect at the animal level (*F*(3, 20) = 6.79, *p* = 0.00245, partial η^2^ = 0.504). Relative to the AD + Sham group, the maximum rising slope was higher by 80.83 mV/ms in the Control group (Tukey-adjusted 95% CI, 16.41 to 145.25; adjusted *p* = 0.0108), by 78.33 mV/ms in the AD + Fer-1 group (95% CI, 13.91 to 142.75; adjusted *p* = 0.0138), and by 92.50 mV/ms in the AD + rTMS group (95% CI, 28.08 to 156.92; adjusted *p* = 0.00346; Figure 8e).

Similarly, the maximum falling slope showed a large overall treatment effect (*F*(3, 20) = 18.46, *p* = 5.58 × 10^−6^, partial η^2^ = 0.735). Compared with the AD + Sham group, the maximum falling slope was more negative in the Control group (mean difference, −80.17 mV/ms; Tukey-adjusted 95% CI, −112.41 to −47.92; adjusted *p* = 5.25 × 10^−6^), the AD + Fer-1 group (mean difference, −51.75 mV/ms; 95% CI, −83.99 to −19.51; adjusted *p* = 0.00118), and the AD + rTMS group (mean difference, −66.17 mV/ms; 95% CI, −98.41 to −33.92; adjusted *p* = 7.02 × 10^−5^; Figure 8f), indicating faster repolarization kinetics than in AD + Sham mice.

Together, these results indicate that APP/PS1 mice exhibited reduced excitability of hippocampal DG granule neurons, characterized by fewer evoked action potentials, a more depolarized AP threshold, a lower AP peak potential, and slower depolarization and repolarization kinetics, whereas resting membrane potential and AP half-width remained unchanged. Both high-frequency rTMS and Fer-1 partially reversed these electrophysiological abnormalities.

#### 3.2.2. Effects of rTMS and Fer-1 on Voltage-Gated Na^+^ Currents

**Figure 9:**
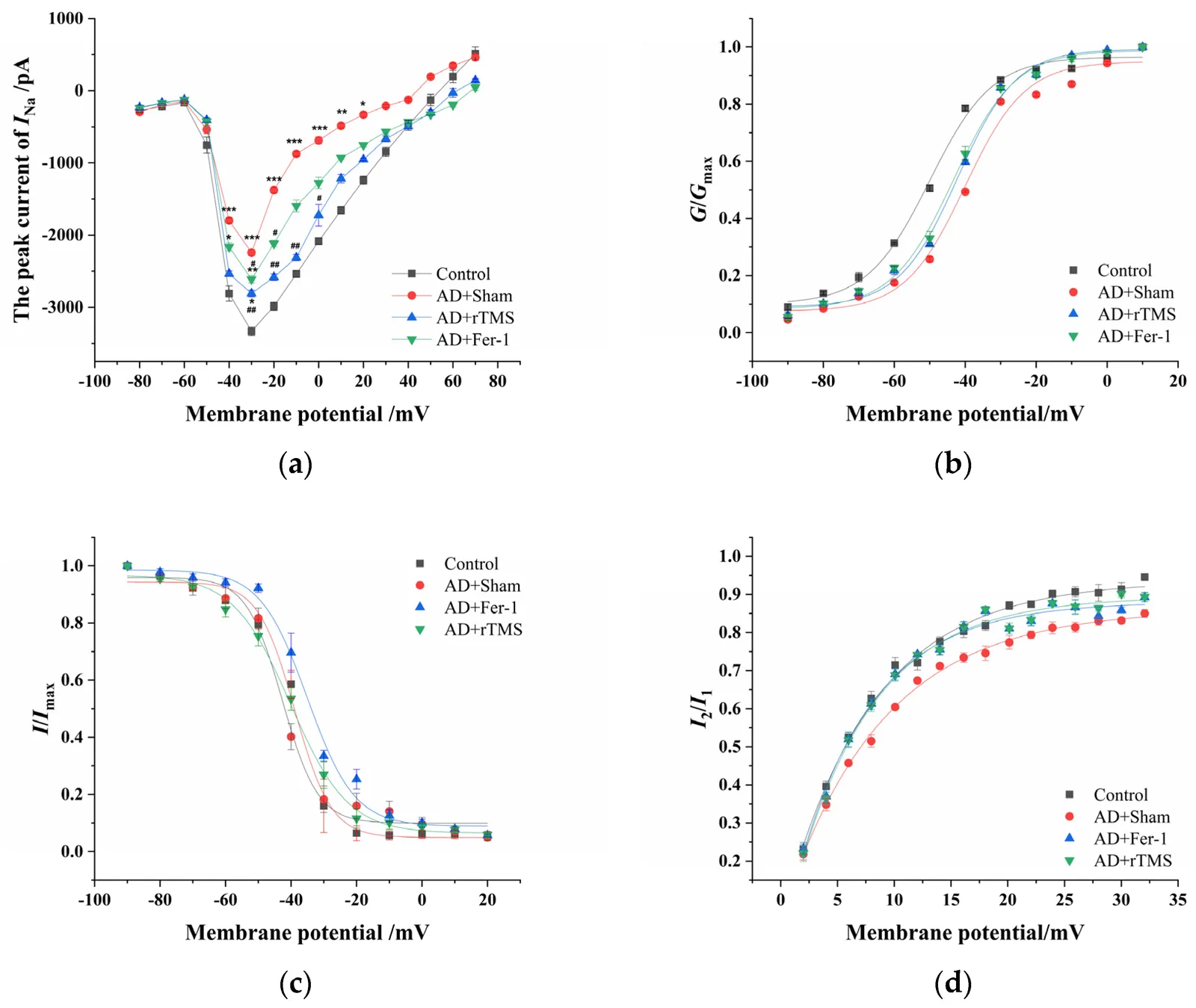
Electrophysiological properties of voltage-gated Na^+^ currents. (**a**) *I*_Na_ amplitude. (**b**) *I*_Na_ activation curves. (**c**) *I*_Na_ inactivation curve. (**d**) Recovery curve from *I*_Na_ inactivation. Data are presented as mean ± SEM from six cells obtained from six mice per group, with one cell recorded from each mouse. For current–voltage relationships, statistical comparisons were performed using two-way repeated-measures ANOVA, with treatment group as the between-subject factor and membrane potential as the within-cell factor, followed by Tukey’s post-hoc test where appropriate. Fitted ion-channel kinetic parameters were analyzed using one-way ANOVA followed by Tukey’s post-hoc test. Significance symbols indicate the between-group comparisons shown in the figure: * *p* < 0.05, ** *p* < 0.01, *** *p* < 0.001 vs. Control; ^#^ *p* < 0.05, ^##^ *p* < 0.01, vs. AD + Sham; *n* = 6.

Voltage-gated Na^+^ currents were recorded from hippocampal DG granule neurons to evaluate whether rTMS and Fer-1 affected Na^+^ channel function in APP/PS1 mice. Na^+^ currents were activated at approximately −50 mV and reached peak inward amplitudes around −30 mV during depolarizing voltage steps (Figure 9a).

For the Na^+^ current–voltage relationship, current amplitudes measured at different test potentials from the same cell were analyzed using two-way repeated-measures ANOVA, with treatment group as the between-subject factor and test membrane potential as the within-cell factor. After Geisser–Greenhouse correction, the analysis identified large effects of treatment group (*F*(3, 20) = 150.21, *p* = 6.97 × 10^−14^, partial η^2^ = 0.958) and test membrane potential (*F*(4.282, 85.64) = 1655.99, *p* = 3.09 × 10^−81^, partial η^2^ = 0.988), together with a treatment group × voltage interaction (*F*(12.85, 85.64) = 37.65, *p* = 1.05 × 10^−29^, partial η^2^ = 0.850).

Because the Na^+^ current reached its maximal inward amplitude at approximately −30 mV, peak inward-current magnitude at this voltage was further compared among groups. Peak current magnitude showed a large overall treatment effect (*F*(3, 20) = 80.27, *p* = 2.51 × 10^−11^, partial η^2^ = 0.923). Compared with the Control group, the AD + Sham group showed a 1089.60 pA smaller peak inward-current magnitude (Tukey-adjusted 95% CI, −1290.27 to −888.94; adjusted *p* = 1.09 × 10^−11^). Relative to AD + Sham, peak inward-current magnitude was increased by 566.68 pA after rTMS (95% CI, 366.01 to 767.34; adjusted *p* = 7.95 × 10^−7^) and by 369.70 pA after Fer-1 treatment (95% CI, 169.04 to 570.36; adjusted *p* = 2.60 × 10^−4^). Nevertheless, peak current magnitude remained lower than Control after rTMS (mean difference, −522.93 pA; 95% CI, −723.59 to −322.26; adjusted *p* = 2.65 × 10^−6^) and Fer-1 treatment (mean difference, −719.90 pA; 95% CI, −920.57 to −519.24; adjusted *p* = 1.69 × 10^−8^). The difference between the AD + rTMS and AD + Fer-1 groups was not statistically significant (mean difference, 196.98 pA; 95% CI, −3.69 to 397.64; adjusted *p* = 0.0555; Figure 9a).

Steady-state activation curves were fitted separately for each recorded cell using the Boltzmann equation to obtain the half-activation voltage (*V*_1/2_) and slope factor (*k*) (Figure 9b; Table 2). Animal-level analysis of the cell-specific fitted parameters showed a large overall group effect on activation *V*_1/2_ (*F*(3, 20) = 16.18, *p* = 1.42 × 10^−5^, partial η^2^ = 0.708). Compared with the Control group, the activation *V*_1/2_ of the AD + Sham group was shifted by 9.95 mV in the depolarizing direction (Tukey-adjusted 95% CI, 5.72 to 14.18; adjusted *p* = 1.20 × 10^−5^). Activation *V*_1/2_ also remained more depolarized than Control after Fer-1 treatment (mean difference, 7.86 mV; 95% CI, 3.62 to 12.09; adjusted *p* = 2.39 × 10^−4^) and rTMS (mean difference, 6.74 mV; 95% CI, 2.51 to 10.97; adjusted *p* = 0.00128). Relative to AD + Sham, *V*_1/2_ shifted numerically in the hyperpolarizing direction by 2.09 mV after Fer-1 treatment (95% CI, −6.33 to 2.14; adjusted *p* = 0.524) and by 3.21 mV after rTMS (95% CI, −7.44 to 1.02; adjusted *p* = 0.180), but neither comparison was statistically significant. The activation slope factor showed little evidence of a group effect (*F*(3, 20) = 0.05, *p* = 0.984, partial η^2^ = 0.008).

Steady-state inactivation curves were fitted separately for each recorded cell using the Boltzmann equation (Figure 9c; Table 2). Animal-level analysis of the cell-specific fitted parameters showed no significant overall group effect on the half-inactivation voltage, *V*_1/2_ (*F*(3, 20) = 1.80, *p* = 0.180, partial η^2^ = 0.212), or the inactivation slope factor, k (*F*(3, 20) = 1.02, *p* = 0.403, partial η^2^ = 0.133). These findings suggest that the steady-state inactivation properties of voltage-gated Na^+^ channels were not markedly altered by APP/PS1 genotype or by either intervention.

Recovery from inactivation was assessed using a paired-pulse protocol (Figure 9d; Table 2). One-way ANOVA revealed a significant group effect on the recovery time constant, τ (*F*(3, 20) = 5.64, *p* = 0.006). Compared with the AD + Sham group, both the AD + rTMS and AD + Fer-1 groups showed lower τ values, consistent with a faster recovery from inactivation after treatment. Post-hoc comparisons indicated that τ was significantly reduced in the AD + Fer-1 group (*p* < 0.01) and AD + rTMS group (*p* < 0.05) compared with the AD + Sham group, whereas no significant difference was observed between the AD + rTMS and AD + Fer-1 groups.

Together, these results suggest that APP/PS1 mice exhibited abnormalities in voltage-gated Na^+^ current amplitude and activation properties in hippocampal DG granule neurons. High-frequency rTMS and Fer-1 partially improved Na^+^ current amplitude abnormalities and accelerated recovery from inactivation, whereas the altered Na^+^ channel activation parameters were not significantly reversed and remained different from Control levels.

#### 3.2.3. Effects of rTMS and Fer-1 on Voltage-Gated K^+^ Currents

Voltage-gated K^+^ currents were examined in hippocampal DG granule neurons to evaluate the effects of rTMS and Fer-1 on K^+^ channel function in APP/PS1 mice. For the *I*_A_ current–voltage relationship, current amplitudes recorded at different test potentials from the same cell were analyzed using two-way repeated-measures ANOVA, with treatment group as the between-subject factor and test membrane potential as the within-cell factor. After Geisser–Greenhouse correction, the analysis showed large effects of treatment group (*F*(3, 20) = 813.98, *p* = 4.61 × 10^−21^, partial η^2^ = 0.992) and test membrane potential (*F*(5.155, 103.09) = 2040.08, *p* = 6.45 × 10^−102^, partial η^2^ = 0.990), together with a treatment group × voltage interaction (*F*(15.464, 103.09) = 31.33, *p* = 5.64 × 10^−32^, partial η^2^ = 0.825).

**Figure 10:**
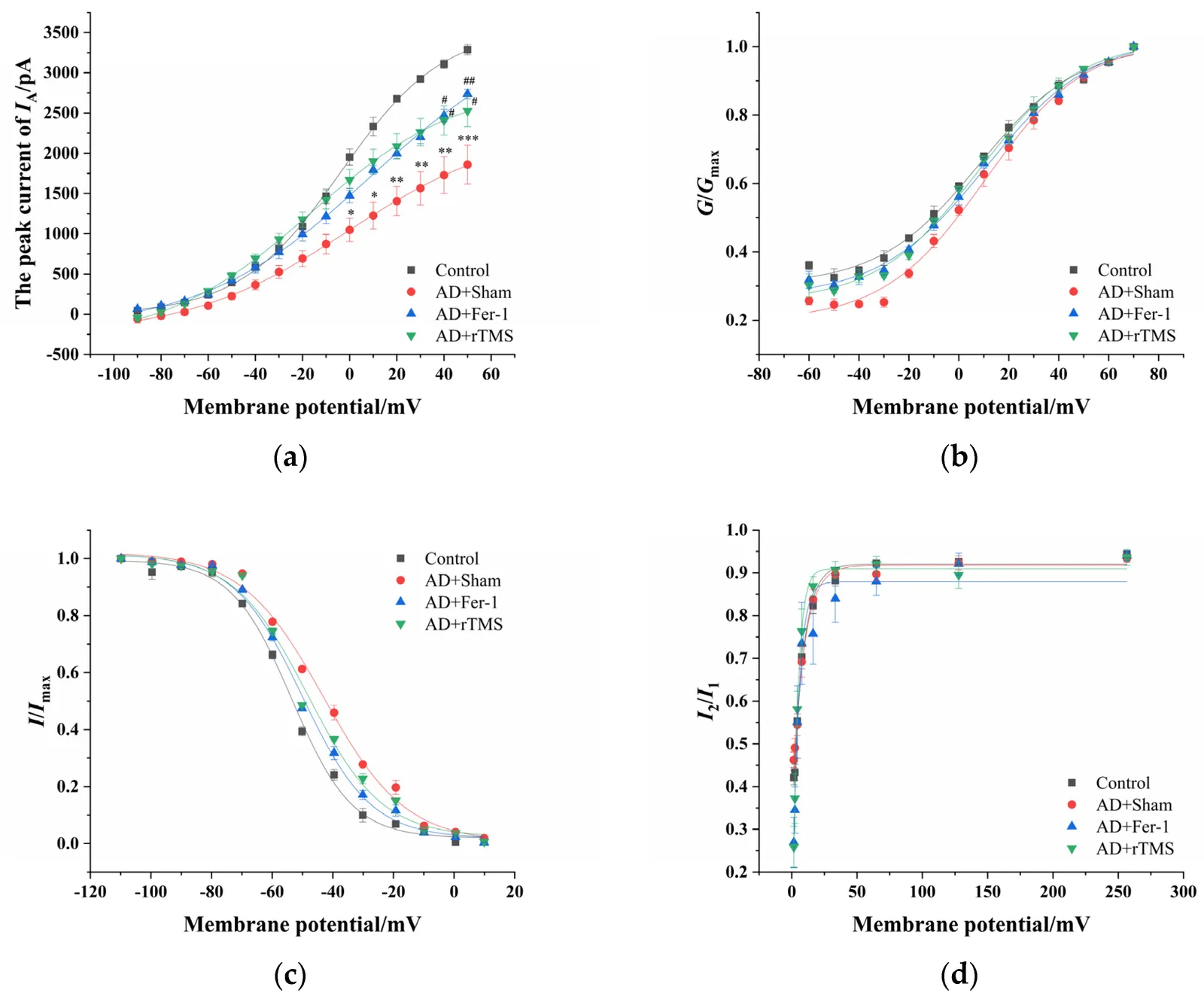
Electrophysiological properties of voltage-gated A-type K^+^ currents. (**a**) *I*_A_ amplitude. (**b**) *I*_A_ activation curves. (**c**) *I*_A_ inactivation curve. (**d**) Recovery curve from *I*_A_ inactivation. Data are presented as mean ± SEM from six cells obtained from six mice per group, with one cell recorded from each mouse. For current–voltage relationships, statistical comparisons were performed using two-way repeated-measures ANOVA, with treatment group as the between-subject factor and membrane potential as the within-cell factor, followed by Tukey’s post-hoc test where appropriate. Fitted ion-channel kinetic parameters were analyzed using one-way ANOVA followed by Tukey’s post-hoc test. Significance symbols indicate the between-group comparisons shown in the figure: * *p* < 0.05, ** *p* < 0.01, *** *p* < 0.001 vs. Control; ^#^ *p* < 0.05, ^##^ *p* < 0.01 vs. AD + Sham; *n* = 6.

Because *I*_A_ reached its maximal amplitude at approximately +50 mV, current amplitude at this voltage was further compared among groups. Peak *I*_A_ amplitude showed a large overall group effect (*F*(3, 20) = 80.17, *p* = 2.54 × 10^−11^, partial η^2^ = 0.923). Compared with the Control group, peak *I*_A_ amplitude was 1428.08 pA lower in the AD + Sham group (Tukey-adjusted 95% CI, −1689.04 to −1167.12; adjusted *p* = 9.46 × 10^−12^). Relative to AD + Sham, peak *I*_A_ amplitude was increased by 877.93 pA after Fer-1 treatment (95% CI, 616.97 to 1138.89; adjusted *p* = 4.92 × 10^−8^) and by 667.42 pA after rTMS (95% CI, 406.46 to 928.37; adjusted *p* = 3.49 × 10^−6^). Nevertheless, peak *I*_A_ amplitude remained lower than Control after Fer-1 treatment (mean difference, −550.15 pA; 95% CI, −811.11 to −289.19; adjusted *p* = 4.98 × 10^−5^) and rTMS (mean difference, −760.67 pA; 95% CI, −1021.62 to −499.71; adjusted *p* = 4.88 × 10^−7^). The difference between the AD + Fer-1 and AD + rTMS groups was not statistically significant (mean difference for AD + rTMS minus AD + Fer-1, −210.51 pA; 95% CI, −471.47 to 50.44; adjusted *p* = 0.142; Figure 10a).

The activation parameters of *I*_A_ were obtained by fitting the activation curve separately for each recorded cell using the Boltzmann equation (Figure 10b; Table 3). Animal-level analysis of the cell-specific fitted parameters showed no significant overall group effect on the *I*_A_ half-activation voltage, *V*_1/2_ (*F*(3, 20) = 0.77, *p* = 0.523, partial η^2^ = 0.104), or activation slope factor, k (*F*(3, 20) = 0.08, *p* = 0.968, partial η^2^ = 0.013). These results indicate that the reduction in *I*_A_ amplitude in AD + Sham neurons was not accompanied by a marked alteration in the voltage dependence of *I*_A_ activation.

The steady-state inactivation parameters of *I*_A_ were obtained by fitting the inactivation curve separately for each recorded cell using the Boltzmann equation (Figure 10c; Table 3). *I*_A_ half-inactivation voltage showed a large overall group effect (*F*(3, 20) = 11.29, *p* = 1.50 × 10^−4^, partial η^2^ = 0.629). Compared with the Control group, the half-inactivation voltage in the AD + Sham group was shifted by 9.92 mV in the depolarizing direction (Tukey-adjusted 95% CI, 5.00 to 14.84; adjusted *p* = 8.70 × 10^−5^). Relative to AD + Sham, the half-inactivation voltage shifted toward more negative potentials by 7.03 mV after Fer-1 treatment (95% CI, −11.95 to −2.11; adjusted *p* = 0.00360) and by 5.17 mV after rTMS (95% CI, −10.09 to −0.25; adjusted *p* = 0.0371). The difference between the AD + Fer-1 and AD + rTMS groups was not statistically significant (mean difference, −1.86 mV; 95% CI, −6.78 to 3.06; adjusted *p* = 0.718). The *I*_A_ inactivation slope factor did not differ significantly among groups (*F*(3, 20) = 0.77, *p* = 0.524, partial η^2^ = 0.103).

Recovery from inactivation was assessed using a paired-pulse protocol, and the recovery curve was fitted separately for each recorded cell to obtain the recovery time constant, τ (Figure 10d; Table 3). Animal-level analysis showed no significant overall group effect on τ (*F*(3, 20) = 0.41, *p* = 0.748, partial η^2^ = 0.058), indicating that the recovery kinetics of *I*_A_ were not markedly altered by APP/PS1 genotype or either intervention. Together, these findings indicate that *I*_A_ activation parameters, inactivation slope, and recovery kinetics remained largely unchanged, whereas the depolarizing shift in *I*_A_ half-inactivation voltage observed in AD + Sham neurons was partially normalized by Fer-1 and rTMS.

**Figure 11:**
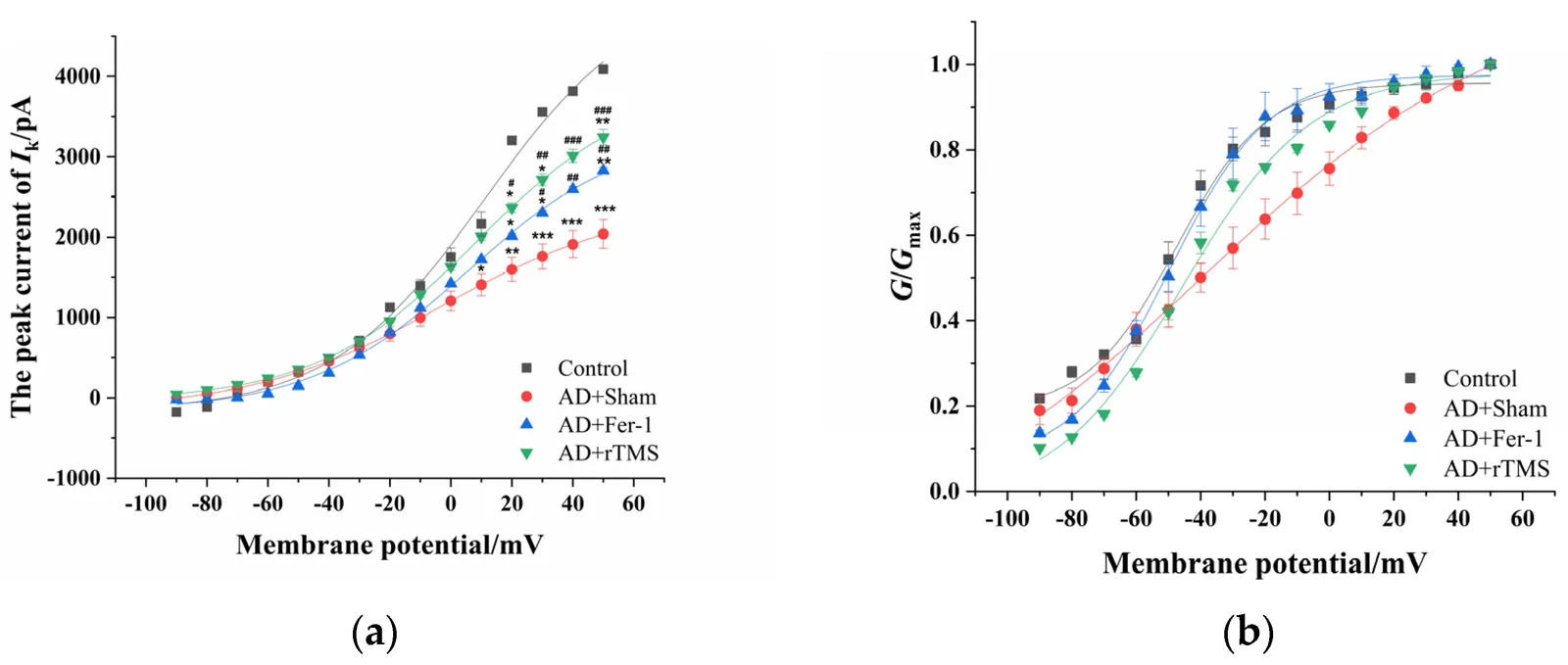
Electrophysiological properties of voltage-gated delayed rectifier K^+^ currents. (**a**) *I*_K_ amplitude. (**b**) *I*_K_ activation curves. Data are presented as mean ± SEM from six cells obtained from six mice per group, with one cell recorded from each mouse. For current–voltage relationships, statistical comparisons were performed using two-way repeated-measures ANOVA, with treatment group as the between-subject factor and membrane potential as the within-cell factor, followed by Tukey’s post-hoc test where appropriate. Fitted ion-channel activation parameters were analyzed using one-way ANOVA followed by Tukey’s post-hoc test. Significance symbols indicate the between-group comparisons shown in the figure: * *p* < 0.05, ** *p* < 0.01, *** *p* < 0.001 vs. Control; ^#^ *p* < 0.05, ^##^ *p* < 0.01, ^###^ *p* < 0.001 vs. AD + Sham; *n* = 6.

For the *I*_K_ current–voltage relationship (Figure 11a), current amplitudes measured at different test potentials from the same cell were analyzed using two-way repeated-measures ANOVA, with treatment group as the between-subject factor and test membrane potential as the within-cell factor. After Geisser–Greenhouse correction, the analysis showed large effects of treatment group (*F*(3, 20) = 1234.02, *p* = 7.41 × 10^−23^, partial η^2^ = 0.995) and test membrane potential (*F*(4.788, 95.751) = 6633.35, *p* = 3.32 × 10^−119^, partial η^2^ = 0.997), together with a treatment group × voltage interaction (*F*(14.363, 95.751) = 159.80, *p* = 3.66 × 10^−60^, partial η^2^ = 0.960).

Because *I*_K_ reached its maximal amplitude at +50 mV, current amplitude at this voltage was further compared among groups. Peak *I*_K_ amplitude showed a large overall group effect (*F*(3,20) = 403.80, *p* = 4.69 × 10^−18^, partial η^2^ = 0.984). Compared with the Control group, peak *I*_K_ amplitude was 2049.09 pA lower in the AD + Sham group (Tukey-adjusted 95% CI, −2217.21 to −1880.97; adjusted *p* = 2.11 × 10^−15^). Relative to AD + Sham, peak *I*_K_ amplitude was increased by 790.37 pA after Fer-1 treatment (95% CI, 622.25 to 958.49; adjusted *p* = 1.51 × 10^−10^) and by 1202.95 pA after rTMS (95% CI, 1034.83 to 1371.07; adjusted *p* = 6.32 × 10^−14^). Nevertheless, peak *I*_K_ amplitude remained lower than Control after Fer-1 treatment (mean difference, −1258.72 pA; 95% CI, −1426.84 to −1090.60; adjusted *p* = 2.79 × 10^−14^) and rTMS (mean difference, −846.14 pA; 95% CI, −1014.26 to −678.02; adjusted *p* = 4.40 × 10^−11^). Peak *I*_K_ amplitude was 412.58 pA higher in the AD + rTMS group than in the AD + Fer-1 group (95% CI, 244.46 to 580.70; adjusted *p* = 6.33 × 10^−6^; Figure 11a).

The activation parameters of *I*_K_ were obtained by fitting the activation curve separately for each recorded cell using the Boltzmann equation (Figure 11b; Table 4). Animal-level analysis of the cell-specific fitted parameters showed no significant overall group effect on the *I*_K_ half-activation voltage, *V*_1/2_ (*F*(3, 20) = 1.99, *p* = 0.149, partial η^2^ = 0.230), or activation slope factor, *k* (*F*(3, 20) = 0.53, *p* = 0.665, partial η^2^ = 0.074). Although activation *V*_1/2_ was numerically more depolarized in the AD + Sham group, the between-group differences were not statistically significant. These findings indicate that *I*_K_ activation voltage dependence was not markedly altered by APP/PS1 genotype or either intervention.

Together, these results indicate that APP/PS1 mice exhibited voltage-gated K^+^ channel abnormalities in hippocampal DG granule neurons, characterized by reduced *I*_A_ and *I*_K_ amplitudes and a depolarizing shift in *I*_A_ steady-state inactivation, whereas the activation voltage dependence of *I*_A_ and *I*_K_ was not markedly altered. High-frequency rTMS and Fer-1 partially ameliorated the reductions in K^+^ current amplitudes and the abnormal shift in *I*_A_ inactivation.

### 3.3. Effects of rTMS and Fer-1 on Ferroptosis-Related Oxidative Injury in Hippocampal Tissue

#### 3.3.1. Effects of rTMS and Fer-1 on Ferroptosis-Related Oxidative Injury Markers

**Figure 12:**
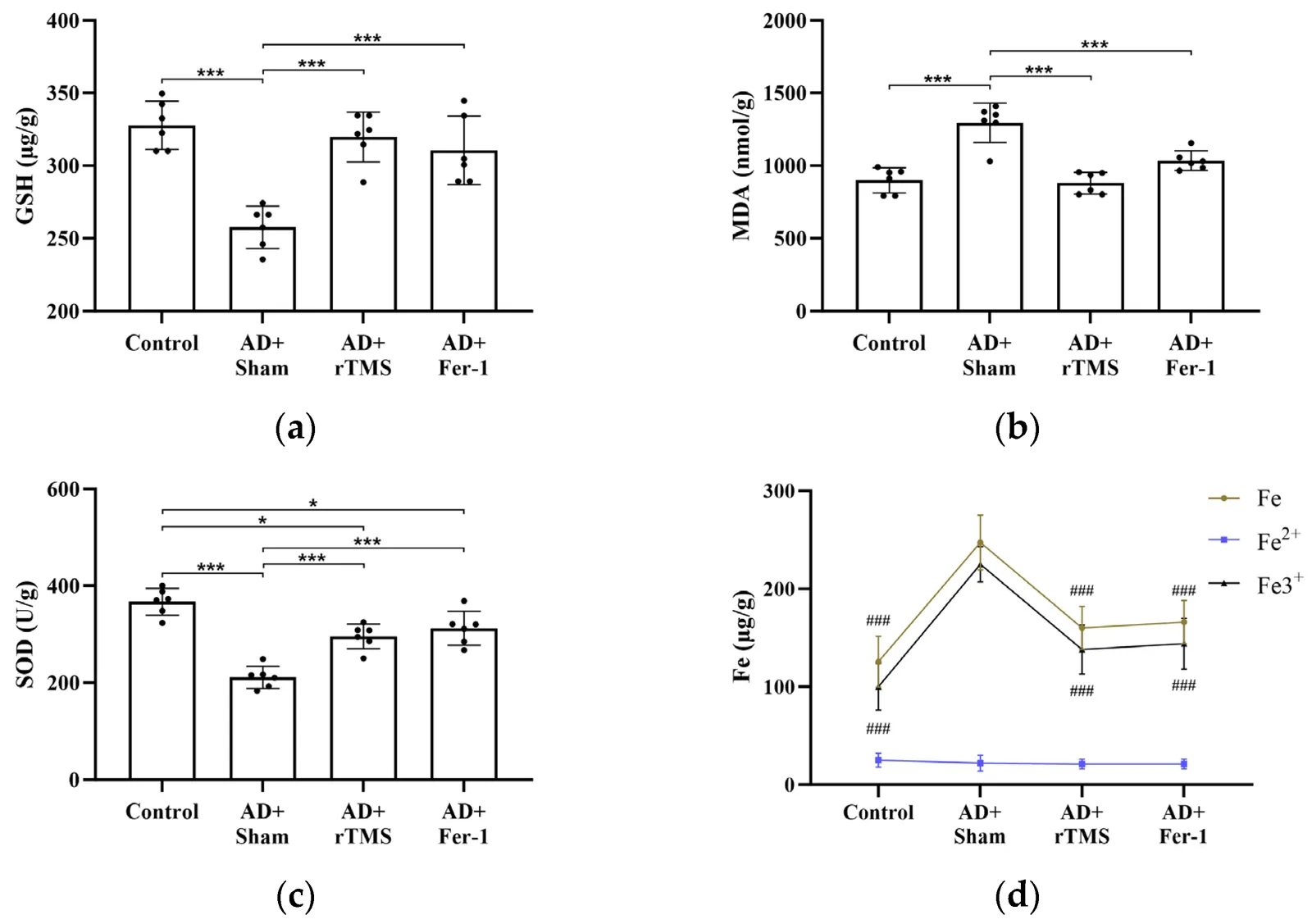
Ferroptosis-related oxidative injury markers in the hippocampus. (**a**) Hippocampal glutathione content. (**b**) Hippocampal malondialdehyde content. (**c**) Hippocampal superoxide dismutase activity. (**d**) Hippocampal total iron content. Data are presented as mean ± SEM from six mice per group, with each mouse contributing one biological sample and the mouse treated as the experimental unit. Statistical comparisons were performed using one-way ANOVA followed by Tukey’s post-hoc test. Significance symbols indicate the between-group comparisons shown in the figure: * *p* < 0.05, *** *p* < 0.001 vs. Control; ^###^ *p* < 0.001 vs. AD + Sham; *n* = 6.

Biochemical assays were performed to evaluate ferroptosis-related oxidative injury markers in the hippocampus. Hippocampal GSH levels showed a large overall group effect (*F*(3, 20) = 18.08, *p* = 6.47 × 10^−6^, partial η^2^ = 0.731). Compared with the Control group, GSH levels were 70.22 units lower in the AD + Sham group (Tukey-adjusted 95% CI, −99.70 to −40.73; adjusted *p* = 9.66 × 10^−6^), indicating impaired antioxidant capacity in APP/PS1 mice. Relative to AD + Sham, GSH levels were increased by 62.13 units after rTMS (95% CI, 32.65 to 91.62; adjusted *p* = 5.00 × 10^−5^) and by 52.85 units after Fer-1 treatment (95% CI, 23.37 to 82.33; adjusted *p* = 3.56 × 10^−4^). Neither the AD + rTMS nor the AD + Fer-1 group differed significantly from the Control group (adjusted *p* = 0.868 and 0.376, respectively; Figure 12a).

Hippocampal MDA levels also showed a large overall group effect (*F*(3, 20) = 24.37, *p* = 6.94 × 10^−7^, partial η^2^ = 0.785). Compared with the Control group, MDA levels were 395.33 units higher in the AD + Sham group (Tukey-adjusted 95% CI, 241.96 to 548.71; adjusted *p* = 3.12 × 10^−6^), consistent with increased lipid peroxidation. Relative to AD + Sham, MDA levels were reduced by 415.17 units after rTMS (95% CI, −568.54 to −261.79; adjusted *p* = 1.51 × 10^−6^) and by 259.00 units after Fer-1 treatment (95% CI, −412.38 to −105.62; adjusted *p* = 6.90 × 10^−4^). MDA levels did not differ significantly from Control after rTMS or Fer-1 treatment (adjusted *p* = 0.983 and 0.0926, respectively). MDA levels were 156.17 units higher in the AD + Fer-1 group than in the AD + rTMS group (95% CI, 2.79 to 309.54; adjusted *p* = 0.0450; Figure 12b).

Hippocampal SOD activity showed a large overall group effect (*F*(3, 20) = 31.51, *p* = 8.95 × 10^−8^, partial η^2^ = 0.825). Compared with the Control group, SOD activity was 155.80 units lower in the AD + Sham group (Tukey-adjusted 95% CI, −201.30 to −110.30; adjusted *p* = 3.68 × 10^−8^). Relative to AD + Sham, SOD activity was increased by 84.07 units after rTMS (95% CI, 38.57 to 129.57; adjusted *p* = 2.52 × 10^−4^) and by 100.87 units after Fer-1 treatment (95% CI, 55.37 to 146.37; adjusted *p* = 2.57 × 10^−5^). Nevertheless, SOD activity remained lower than Control after rTMS (mean difference, −71.73 units; 95% CI, −117.23 to −26.23; adjusted *p* = 0.00141) and Fer-1 treatment (mean difference, −54.93 units; 95% CI, −100.43 to −9.43; adjusted *p* = 0.0145). The AD + rTMS and AD + Fer-1 groups did not differ significantly (mean difference, −16.80 units; 95% CI, −62.30 to 28.70; adjusted *p* = 0.732; Figure 12c).

Total iron levels showed a large overall group effect (*F*(3, 20) = 26.26, *p* = 3.87 × 10^−7^, partial η^2^ = 0.798). Compared with the Control group, total iron was increased by 121.70 μg/g in the AD + Sham group (Tukey-adjusted 95% CI, 81.89 to 161.51; adjusted *p* = 2.31 × 10^−7^). Relative to AD + Sham, total iron was reduced by 87.20 μg/g after rTMS (95% CI, −127.01 to −47.39; adjusted *p* = 3.02 × 10^−5^) and by 81.20 μg/g after Fer-1 treatment (95% CI, −121.01 to −41.39; adjusted *p* = 7.59 × 10^−5^). Total iron levels in the AD + rTMS group did not differ significantly from those in the Control group (mean difference, 34.50 μg/g; 95% CI, −5.31 to 74.31; adjusted *p* = 0.104), whereas levels in the AD + Fer-1 group remained modestly higher than Control (mean difference, 40.50 μg/g; 95% CI, 0.69 to 80.31; adjusted *p* = 0.0453; Figure 12d). These findings indicate that both rTMS and Fer-1 attenuated hippocampal iron accumulation in APP/PS1 mice, with a more complete normalization observed after rTMS.

Together, these results indicate that APP/PS1 mice exhibited hippocampal oxidative injury characterized by GSH depletion, increased lipid peroxidation, reduced antioxidant enzyme activity, and elevated total iron levels. High-frequency rTMS was associated with biochemical changes consistent with attenuation of ferroptosis-related oxidative injury in the hippocampus. Fer-1 produced broadly similar changes, although the extent of normalization varied across individual markers.

#### 3.3.2. Effects of rTMS and Fer-1 on Mitochondrial Ultrastructure

**Figure 13:**
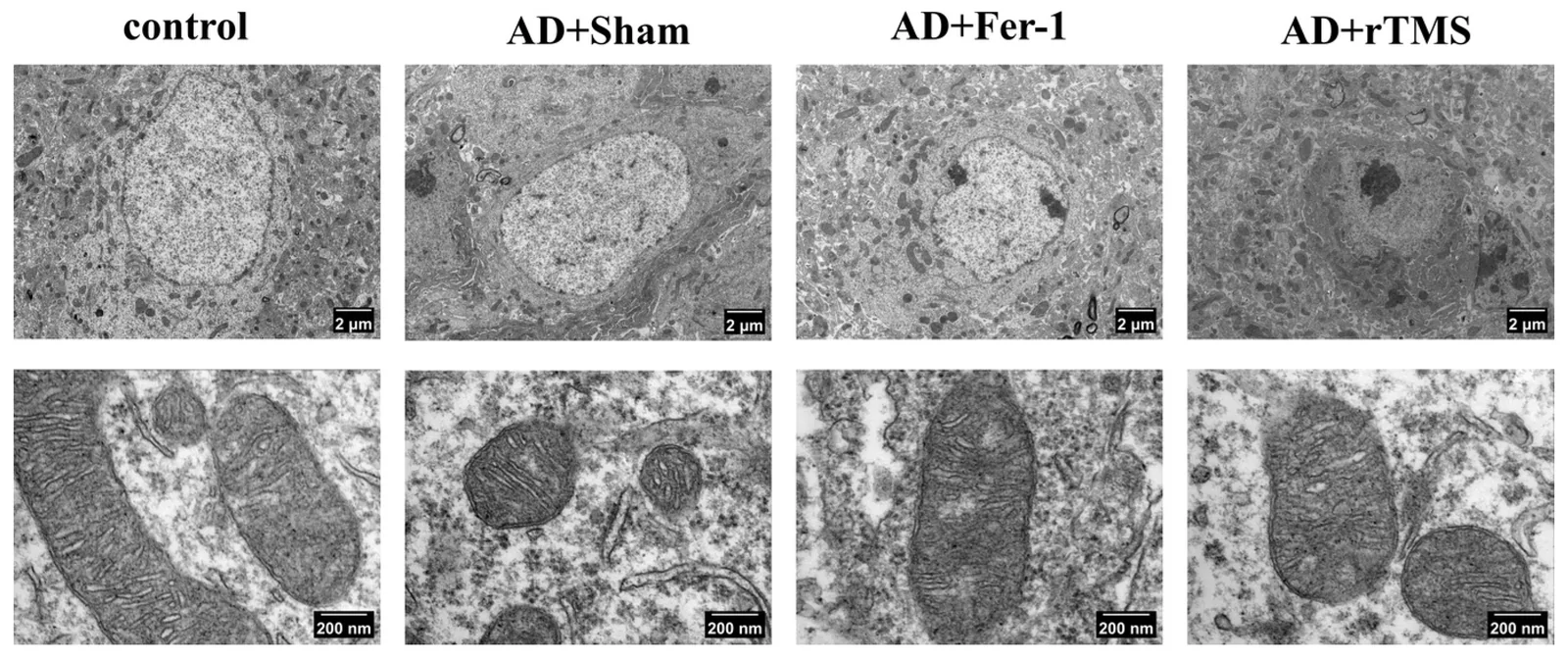
TEM images of perinuclear mitochondria in hippocampal neurons. Representative images are shown from an independent TEM cohort of three mice per group. Brightness and contrast adjustments for presentation were applied uniformly across all representative TEM images and did not affect the quantitative morphometric analysis.

Transmission electron microscopy was used to examine mitochondrial ultrastructure in hippocampal neurons. Compared with the Control group, hippocampal neurons from the AD + Sham group showed obvious mitochondrial abnormalities, including mitochondrial shrinkage, condensed matrix, and disrupted or reduced cristae (Figure 13). These ultrastructural changes were compatible with mitochondrial injury associated with ferroptosis-related oxidative damage, although they are not specific to ferroptosis. In contrast, mitochondria in the AD + rTMS and AD + Fer-1 groups showed relatively preserved morphology, with less prominent shrinkage and better cristae organization.

**Figure 14:**
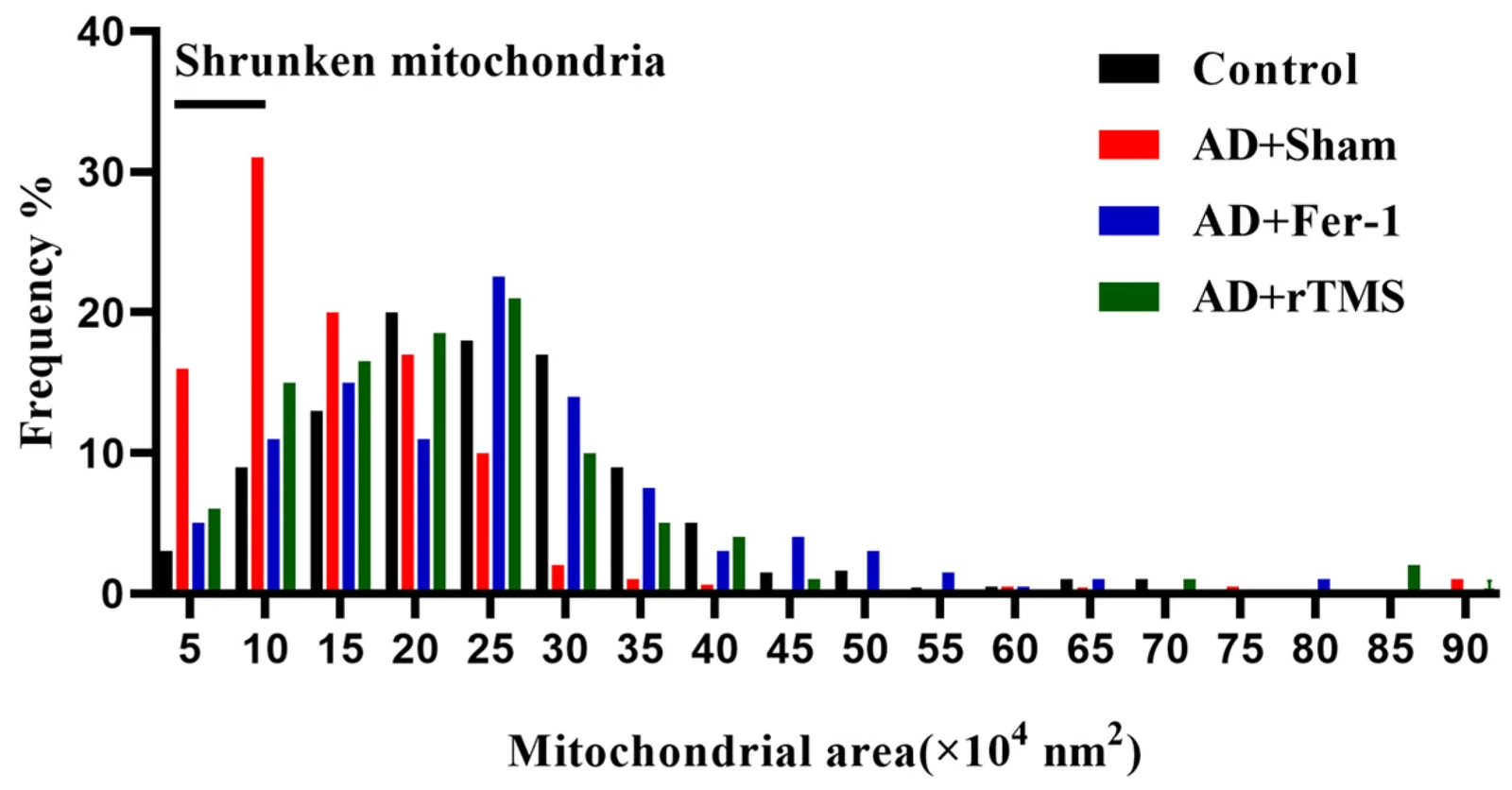
Frequency distribution of mitochondrial area in the perinuclear region. Mitochondrial cross-sectional areas were measured from ten non-overlapping TEM images per mouse in an independent cohort of three mice per group. The distributions are presented descriptively; individual mitochondrial profiles, images, and area bins were not treated as independent experimental units for inferential statistical analysis.

To quantify mitochondrial alterations, perinuclear mitochondria were analyzed in hippocampal neurons. Three mice were included in each group, and 10 fields were analyzed per animal, yielding 30 micrographs per group. At least four mitochondria were measured in each image, resulting in more than 120 mitochondrial cross-sections per group for morphometric assessment (Figure 14). For statistical analysis, animal-level values were used to reduce pseudo-replication.

Descriptive frequency distributions showed that mitochondrial cross-sectional areas in the AD + Sham group were shifted toward smaller values compared with the Control group. The distributions in the AD + rTMS and AD + Fer-1 groups appeared closer to that of the Control group. Because individual mitochondrial profiles were nested within images and animals, these group-level frequency distributions were interpreted descriptively and were not subjected to inferential statistical testing.

**Figure 15:**
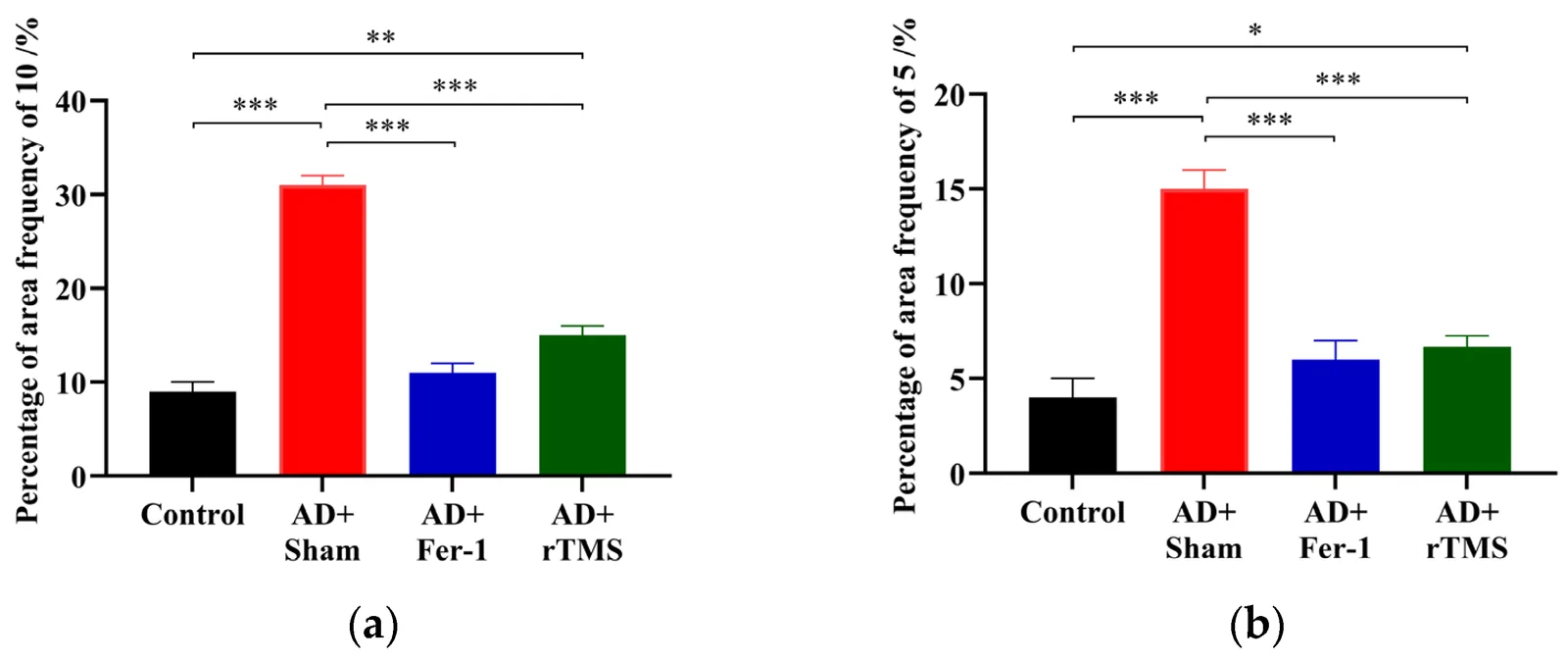
Quantitative analysis of mitochondrial atrophy in the perinuclear region. (**a**) Percentage of mitochondria with an area < 10 μm^2^. (**b**) Percentage of mitochondria with an area < 5 μm^2^. For each mouse, measurements from ten TEM images were combined to generate one animal-level percentage. Data are presented as mean ± SEM from three mice per group, with the mouse treated as the experimental unit. Statistical comparisons were performed using one-way ANOVA followed by Tukey’s post-hoc test. Significance symbols indicate the Tukey-adjusted between-group comparisons shown in the figure: * *p* < 0.05, ** *p* < 0.01, *** *p* < 0.001, *n* = 3.

Animal-level analysis showed a significant group effect on the percentage of mitochondrial profiles with a cross-sectional area < 10 μm^2^ (*F*(3, 8) = 299.00, *p* = 1.50 × 10^−8^, partial η^2^ = 0.991). Compared with the Control group, the AD + Sham group showed a 22.00-percentage-point higher proportion of mitochondrial profiles below this threshold (Tukey-adjusted 95% CI, 19.39 to 24.61; adjusted *p* = 1.84 × 10^−8^). Relative to AD + Sham, this proportion was reduced by 20.00 percentage points in the AD + Fer-1 group (95% CI, −22.61 to −17.39; adjusted *p* = 3.91 × 10^−8^) and by 16.00 percentage points in the AD + rTMS group (95% CI, −18.61 to −13.39; adjusted *p* = 2.26 × 10^−7^). The AD + Fer-1 group did not differ significantly from the Control group (mean difference, 2.00 percentage points; 95% CI, −0.61 to 4.61; adjusted *p* = 0.144), whereas the AD + rTMS group remained higher than the Control group (mean difference, 6.00 percentage points; 95% CI, 3.39 to 8.61; adjusted *p* = 3.67 × 10^−4^) and the AD + Fer-1 group (mean difference, 4.00 percentage points; 95% CI, 1.39 to 6.61; adjusted *p* = 0.00523; Figure 15a).

Similarly, the animal-level percentage of mitochondrial profiles with a cross-sectional area < 5 μm^2^ differed significantly among groups (*F*(3, 8) = 84.90, *p* = 2.09 × 10^−6^, partial η^2^ = 0.970). Compared with the Control group, the AD + Sham group showed an 11.00-percentage-point higher proportion of mitochondrial profiles below this threshold (Tukey-adjusted 95% CI, 8.61 to 13.39; adjusted *p* = 2.06 × 10^−6^). Relative to AD + Sham, this proportion was reduced by 9.00 percentage points in the AD + Fer-1 group (95% CI, −11.39 to −6.61; adjusted *p* = 9.59 × 10^−6^) and by 8.33 percentage points in the AD + rTMS group (95% CI, −10.72 to −5.95; adjusted *p* = 1.72 × 10^−5^). The AD + Fer-1 group did not differ significantly from the Control group (mean difference, 2.00 percentage points; 95% CI, −0.39 to 4.39; adjusted *p* = 0.104), whereas the AD + rTMS group remained slightly higher than the Control group (mean difference, 2.67 percentage points; 95% CI, 0.28 to 5.05; adjusted *p* = 0.0296). The AD + rTMS and AD + Fer-1 groups did not differ significantly (mean difference, 0.67 percentage points; 95% CI, −1.72 to 3.05; adjusted *p* = 0.808; Figure 15b).

These ultrastructural findings indicate that APP/PS1 mice exhibited hippocampal mitochondrial abnormalities characterized by mitochondrial shrinkage and increased mitochondrial atrophy. High-frequency rTMS treatment was accompanied by reduced mitochondrial atrophy and improved mitochondrial ultrastructure, although the morphometric recovery remained partial. Fer-1 showed a similar protective profile and produced a greater shift toward Control-level values in mitochondrial atrophy-related frequency measures.

## 4. Discussion

In the present study, high-frequency rTMS improved cognitive performance, partially restored hippocampal DG granule cell excitability and voltage-gated Na^+^ and K^+^ current abnormalities, and was accompanied by improvements in biochemical and mitochondrial indicators compatible with ferroptosis-related injury in APP/PS1 mice. Fer-1 produced partially overlapping, but not identical, effects across behavioral, electrophysiological, biochemical, and ultrastructural measures. Together, these results indicate that functional improvement after rTMS was associated with reduced ferroptosis-related oxidative injury in the hippocampus. Because mechanistic causality was not directly tested, this association should not be interpreted as definitive evidence that rTMS acts through direct ferroptosis inhibition.

The behavioral results showed that APP/PS1 mice exhibited deficits in object recognition and passive avoidance performance, both of which were improved by high-frequency rTMS and Fer-1 treatment. Cognitive dysfunction in AD is closely associated with hippocampal network disruption, abnormal neuronal activity, and impaired information processing. Previous studies have shown that network abnormalities and altered excitability are important contributors to cognitive impairment in AD, and epileptiform or hyperexcitable network activity may occur during the disease process [26,27,28]. Although the present study focused on DG granule neurons rather than whole-brain network activity, the behavioral improvements observed after rTMS are consistent with a contribution of restored hippocampal neuronal function to cognitive performance.

At the cellular level, APP/PS1 mice showed reduced evoked action potential firing in hippocampal DG granule neurons, together with elevated AP threshold, reduced AP peak potential, and slower depolarization and repolarization kinetics. These changes indicate a reduction in intrinsic neuronal excitability. The generation and shape of action potentials depend on the coordinated activation and inactivation of voltage-gated Na^+^ and K^+^ channels. Na^+^ channels mainly drive the rapid depolarizing phase and determine AP threshold and peak amplitude, whereas K^+^ channels contribute to repolarization, firing pattern, and membrane excitability [29,30]. Thus, the reduced AP output and altered AP waveform in AD + Sham neurons are consistent with impaired voltage-gated ion-channel function. Both rTMS and Fer-1 increased AP firing and improved several AP parameters, indicating partial restoration of DG granule cell excitability.

The ion-channel results further support this interpretation. APP/PS1 mice showed reduced voltage-gated Na^+^ current amplitude, and both rTMS and Fer-1 increased Na^+^ current amplitude compared with AD + Sham. However, Na^+^ current amplitudes and activation parameters were not fully restored to Control levels, indicating partial recovery. Na^+^ channel activation _1/2_ in AD-related groups remained shifted in the depolarizing direction compared with Control, suggesting persistent alteration of voltage-dependent gating. For K^+^ currents, both *I*_A_ and *I*_K_ amplitudes were reduced in AD + Sham neurons. This reduction may contribute to slower repolarization kinetics, as reflected by the altered maximum falling slope of APs. Meanwhile, the dominant reduction in Na^+^ current likely contributed to reduced AP firing despite the decrease in outward K^+^ currents. Overall, this pattern suggests that neuronal dysfunction in APP/PS1 mice involves broad impairment of voltage-gated ion-channel function rather than alteration of a single current component.

Activity-dependent plasticity of intrinsic excitability is an important mechanism by which neurons adjust their firing properties, and alterations in ion-channel function can strongly affect neuronal output [31]. The ion-channel changes observed after rTMS may arise through more than one mechanism. They may reflect activity-dependent plasticity induced by altered neuronal activity and voltage-gated channel function, while improved redox homeostasis may also preserve membrane and ion-channel function by reducing oxidative and lipid-peroxidative injury. These mechanisms are not mutually exclusive, and the present study cannot determine their relative contributions to electrophysiological recovery.

An important finding of this study is that Fer-1, a pharmacological comparator targeting lipid peroxidation associated with ferroptosis-related injury, produced partially similar electrophysiological effects to rTMS. However, this similarity does not imply that the two interventions act through the same molecular pathway. Fer-1 primarily limits lipid-peroxidation-related injury, whereas rTMS is a neuromodulatory intervention capable of affecting neuronal activity and plasticity through broader mechanisms. The two interventions may therefore act through distinct upstream processes that converge on reduced oxidative injury, preservation of membrane and ion-channel function, and improved neuronal output. Lipid peroxidation can alter membrane properties and cation handling, thereby affecting ion-channel function and membrane excitability [32]. Accordingly, the overlapping electrophysiological effects support phenotypic convergence between rTMS and Fer-1, but not a shared causal mechanism.

The biochemical results showed a pattern of oxidative and iron-related alterations compatible with ferroptosis-related injury in the hippocampus of APP/PS1 mice. GSH depletion, lipid peroxidation, oxidative stress, and iron accumulation are important features associated with ferroptosis and neurodegenerative injury [33,34]. Both rTMS and Fer-1 increased GSH, reduced MDA and total iron levels, and partially improved SOD activity. Notably, SOD activity remained lower than Control levels in both treatment groups, indicating incomplete antioxidant recovery. Thus, rTMS was associated with improvement in biochemical indices consistent with attenuation of ferroptosis-related oxidative injury, although recovery was incomplete.

The TEM results showed mitochondrial shrinkage, condensed matrix, and disrupted or reduced cristae in hippocampal neurons from AD + Sham mice. Animal-level morphometric analysis further showed increased proportions of mitochondrial profiles with small cross-sectional areas. Mitochondrial shrinkage and reduced cristae have been described in association with ferroptosis-related injury, but these abnormalities are not specific evidence of ferroptotic cell death [35]. Both rTMS and Fer-1 reduced the proportion of small mitochondrial profiles, although the degree of recovery differed between the interventions. Because mitochondrial cross-sectional area is a two-dimensional measurement influenced by sectioning orientation, it should not be interpreted as a direct measure of mitochondrial volume. Overall, these findings are compatible with reduced mitochondrial injury after treatment but do not establish a ferroptosis-specific mechanism.

Taken together, the multi-level findings show a coherent pattern of functional and cellular improvement after high-frequency rTMS. At the behavioral level, rTMS improved recognition and passive avoidance performance in APP/PS1 mice. At the electrophysiological level, it increased DG granule cell excitability and partially restored voltage-gated Na^+^ and K^+^ current abnormalities. These changes were accompanied by reduced oxidative injury, lower total iron accumulation, and improved mitochondrial morphology. Fer-1 produced partially overlapping effects, but this convergence does not establish a shared upstream mechanism. A recent study in aging-related cognitive impairment similarly reported that high-frequency rTMS improved cognitive function while reducing oxidative stress and ferroptosis-related alterations [36]. Thus, the present findings support an association between rTMS-induced functional improvement and attenuation of ferroptosis-related oxidative injury, but do not establish ferroptosis inhibition as a necessary or sufficient mechanism.

The translational relevance of these findings should be interpreted cautiously. The stimulation paradigm used in this mouse study differs from clinical rTMS in stimulation parameters, coil-to-brain geometry, magnetic-field distribution, disease stage, and outcome measures. In particular, because the 56-mm circular coil was large relative to the mouse head, stimulation was broadly distributed rather than focally targeted to a cortical region. In addition, six-month-old APP/PS1 mice do not reproduce the full biological and clinical heterogeneity of Alzheimer’s disease in patients. Therefore, the present findings should not be directly extrapolated to therapeutic efficacy in patients. Rather, they should be regarded as preclinical evidence supporting further mechanistic investigation and subsequent translational validation.

This study has several limitations. First, all experiments were performed exclusively in female APP/PS1 mice. The absence of male animals limits the generalizability of the findings across sexes. In addition, the estrous cycle was not monitored or controlled, and associated hormonal variation may have contributed to variability in behavioral, electrophysiological, and biochemical outcomes. Future studies should include both female and male animals and prospectively monitor or control for estrous-cycle stage when evaluating sex-dependent treatment effects.

Second, no a priori sample-size calculation was performed, and the study was designed as an exploratory preclinical investigation. The sample size of six mice per group limited power to detect small-to-moderate effects, while the independent TEM cohort of three mice per group should be regarded as exploratory for quantitative morphometric analysis. The small group sizes also limit the reliability of normality and homogeneity-of-variance testing. Accordingly, nonsignificant findings should not be interpreted as evidence of equivalence or absence of an effect. In addition, the identities and positions of the novel objects in the NOR test were not counterbalanced, and no preliminary object-preference test was performed. Although total exploration time did not differ among groups, effects of innate object or position preference cannot be completely excluded.

Third, electrophysiological recordings were limited to hippocampal DG granule neurons and therefore do not capture circuit-level changes in other brain regions. Because the 56-mm circular coil was large relative to the mouse head, stimulation was broadly distributed and non-focal; thus, the hippocampal changes should not be interpreted as evidence of direct or selective hippocampal stimulation. Electric-field modeling and region-specific validation were not performed. The sham procedure reproduced the auditory component of stimulation but may not have fully reproduced cutaneous sensation or mechanical vibration, and residual magnetic-field exposure at the head was not directly measured. Moreover, other mechanisms potentially contributing to rTMS effects were not directly examined. Therefore, ferroptosis-related oxidative injury should be considered one component of the observed response rather than the sole biological explanation.

Fourth, the biochemical and ultrastructural indicators examined in this study, including GSH, MDA, SOD, iron-related indices, and mitochondrial morphology, reflect ferroptosis-related oxidative and mitochondrial injury but are not specific markers of ferroptosis. The study did not directly assess lipid reactive oxygen species, GPX4 activity, or key ferroptosis-related proteins such as SLC7A11, ACSL4, and FSP1, nor did it include ferroptosis-specific genetic or rescue interventions. Therefore, further studies incorporating pathway-specific molecular and interventional approaches are needed to determine whether ferroptosis is causally involved in the effects of rTMS.

An additional statistical limitation is the large number of behavioral, electrophysiological, biochemical, kinetic, and morphometric outcomes examined in this exploratory study. Although Tukey’s procedure controlled multiple pairwise comparisons within individual endpoints, no global multiplicity correction was applied across the heterogeneous set of outcomes, and an increased overall risk of type I error therefore cannot be excluded. Individual significant findings should consequently be interpreted together with effect sizes, confidence intervals, and the broader pattern of results. In addition, the electrophysiological and TEM datasets had hierarchical sampling structures. Animal-level values were used for inferential analyses to reduce pseudoreplication; however, the small number of animals limited the application of more complex multilevel models.

Finally, Fer-1 was used as a pharmacological comparator targeting ferroptosis-related lipid peroxidation. However, a single pharmacological comparator cannot establish pathway specificity or demonstrate that ferroptosis inhibition is necessary or sufficient for the effects of rTMS. Therefore, the partially overlapping effects of rTMS and Fer-1 should be interpreted as phenotypic similarity rather than evidence of a shared ferroptosis-specific causal mechanism.

## 5. Conclusions

High-frequency rTMS improved cognitive performance and hippocampal neuronal function in female APP/PS1 mice. These improvements were accompanied by reduced oxidative, iron-related, and mitochondrial abnormalities compatible with attenuation of ferroptosis-related oxidative injury. Fer-1 produced partially overlapping, but not identical, effects, consistent with an association between ferroptosis-related oxidative injury and hippocampal neuronal dysfunction rather than a shared causal pathway. Importantly, the present study does not demonstrate that ferroptosis inhibition is either necessary or sufficient for the effects of rTMS. These findings should therefore be regarded as preclinical evidence supporting further mechanistic investigation and should not be directly extrapolated to therapeutic efficacy in patients with Alzheimer’s disease.

## Figures and Tables

**Table 1:** Composition and pH adjustment of solutions used for whole-cell patch-clamp recordings.

Solution Name	Solution Components (m mol/L)	pH & Adjust pH
Cutting solution	MgCl_2_ 6, KCl 2.5, CaCl_2_ 1, NaH_2_PO_4_ 2H_2_O 1.625, glucose 11, NaHCO_3_ 26, sucrose 220	7.4 (HCl, KOH)
ACSF	CaCl_2_ 2, NaCl 124, KCl 3, glucose 11, MgCl_2_ 2, NaH_2_PO_4_·2H_2_O 1.625, HEPES 5, NaHCO_3_ 26	7.4 (HCl, KOH)
Intracellular solution (AP)	MgCl_2_ 2, K-gluconate 125, Na-ATP 3, CaCl_2_ 1, HEPES 10, Na-GTP 0.3, EGTA 11	7.2–7.3 (HCl, KOH)
Extracellular solution (*I*_Na_)	NaCl 130, CdCl_2_ 0.2, KCl 3, CaCl_2_ 2, MgCl_2_ 2, TEA-Cl 25, glucose 10, HEPES 10, 4-AP 3	7.4 (HCl, KOH)
Intracellular solution (*I*_Na_)	NaCl 140, Mg-ATP 2, HEPES 10, MgCl_2_ 2, EGTA 10	7.4 (HCl, CsOH)
Extracellular solution (*I*_K_)	MgCl_2_ 1, glucose 10, TTX 0.001, NaCl 130, CdCl_2_ 0.2, CaCl_2_ 2, HEPES 10, KCl 5, 4-AP 3	7.4 (HCl, NaOH)
Extracellular solution (*I*_A_)	CaCl_2_ 2, NaCl 130, TEA-Cl 25, KCl 5, MgCl_2_ 1, HEPES 10, CdCl_2_ 0.2, glucose 10, TTX 0.001	7.4 (HCl, NaOH)
Intracellular solution (*I*_A_, *I*_K_)	Mg-ATP 2, MgCl_2_ 2, HEPES 10, KCl 140, EGTA 10	7.2 (HCL, KOH)

**Table 2:** Effects of rTMS and Fer-1 on activation, inactivation, and recovery kinetic parameters of voltage-gated Na^+^ currents. Data are presented as mean ± SEM from six cells obtained from six mice per group, with one cell recorded from each mouse. Statistical comparisons were performed using one-way ANOVA followed by Tukey’s post-hoc test. Significance symbols indicate the between-group comparisons shown in the table: * *p* < 0.05, ** *p* < 0.01, *** *p* < 0.001 vs. Control.

	*I*_Na_ Activation Curve (*n* = 6)		*I*_Na_ Inactivation Curve (*n* = 6)		*I*_Na_ Recovery Curve (*n* = 6)
	_1/2_/mV	*k*	_1/2_/mV	*k*	*τ*/ms
Control	−50.01 ± 0.10	8.81 ± 0.91	−38.91 ± 1.92	5.58 ± 0.82	7.90 ± 0.43
AD + Sham	−40.06 ± 1.62 ***	8.46 ± 1.47	−42.67 ± 1.01	5.45 ± 0.88	8.77 ± 0.34
AD + Fer-1	−42.152 ± 0.96 ***	8.26 ± 0.87	−38.02 ± 1.05	7.18 ± 0.93	6.62 ± 0.43 **
AD + rTMS	−43.27 ± 1.01 ***	8.68 ± 0.92	−40.17 ± 1.81	6.73 ± 0.73	6.95 ± 0.43 *

**Table 3:** Effects of rTMS and Fer-1 on activation, inactivation, and recovery kinetic parameters of voltage-gated A-type K^+^ currents. Data are presented as mean ± SEM from six cells obtained from six mice per group, with one cell recorded from each mouse. Statistical comparisons were performed using one-way ANOVA followed by Tukey’s post-hoc test. Significance symbols indicate the between-group comparisons shown in the table: * *p* < 0.05 vs. Control; ^#^ *p* < 0.05, ^##^ *p* < 0.01 vs. AD + Sham; *n* = 6.

	*I*_A_ Activation Curve (*n* = 6)		*I*_A_ Inactivation Curve (*n* = 6)		*I*_A_ Recovery Curve (*n* = 6)
	_1/2_/mV	*k*	_1/2_/mV	*k*	*τ*/ms
Control	8.03 ± 1.85	20.01 ± 2.08	−52.97 ± 0.95	13.38 ± 0.93	8.01 ± 0.85
AD + Sham	10.44 ± 2.14	20.33 ± 2.40	−43.05 ± 1.30 *	13.52 ± 1.25	8.91 ± 0.84
AD + Fer-1	10.90 ± 1.52	21.39 ± 1.74	−50.08 ± 0.96 ^##^	11.32 ± 0.89	7.72 ± 1.05
AD + rTMS	7.83 ± 1.69	20.40 ± 1.93	−48.22 ± 1.63 ^#^	12.21 ± 1.55	8.02 ± 0.26

**Table 4:** Effects of rTMS and Fer-1 on activation, inactivation, and recovery kinetic parameters of voltage-gated delayed rectifier K^+^ currents. Data are presented as mean ± SEM from six cells obtained from six mice per group, with one cell recorded from each mouse. Statistical comparisons were performed using one-way ANOVA followed by Tukey’s post-hoc test. Significance symbols indicate the between-group comparisons shown in the table: *n* = 6.

	*I*_K_ Inactivation Curve (*n* = 6)	
	_1/2_/mV	*k*
Control	−47.52 ± 2.24	13.83 ± 1.88
AD + Sham	−39.00 ± 5.11	17.29 ± 6.53
AD + Fer-1	−49.53 ± 1.36	15.19 ± 1.10
AD + rTMS	−45.75 ± 3.01	I19.96 ± 2.50

## Data Availability

The data presented in this study are available on request from the corresponding author due to ongoing related research and data management restrictions.

## Abbreviations

The following abbreviations are used in this manuscript:

AD	Alzheimer’s disease
APP/PS1	Amyloid precursor protein/presenilin 1
rTMS	Repetitive transcranial magnetic stimulation
Fer-1	Ferrostatin-1
WT	Wild-type
DG	Dentate gyrus
NOR	Novel object recognition
SDT	Step-down test
CI	Cognitive index
AP	Action potential
RMP	Resting membrane potential
MT	Motor threshold
ACSF	Artificial cerebrospinal fluid
TEM	Transmission electron microscopy
GSH	Glutathione
MDA	Malondialdehyde
SOD	Superoxide dismutase
ROS	Reactive oxygen species
